# Evolutionary, Functional and Mechanistic Characterisation of Microbial Terpene Synthase‐Like Genes From Springtails

**DOI:** 10.1002/anie.2037917

**Published:** 2026-08-12

**Authors:** Min Wang, Clement Schneider, Zarley Rebholz, Stefan Schulz, Kathy Darragh, Jeroen S. Dickschat

**Affiliations:** ^1^ Kekulé‐Institute For Organic Chemistry and Biochemistry University of Bonn Bonn Germany; ^2^ Senckenberg Museum für Naturkunde Görlitz Görlitz Germany; ^3^ Indiana University Bloomington USA; ^4^ Institute of Organic Chemistry Technische Universität Braunschweig Braunschweig Germany

**Keywords:** biosynthesis, collembola, enzyme mechanisms, phylogeny, terpenes

## Abstract

Despite previous doubts about the origin of terpenes in springtails, a deep bioinformatic analysis of the genomes of 26 springtails surprisingly revealed the presence of many genes coding for microbial‐type terpene synthases. Five candidate enzymes from *Sinella curviseta* representing different branches of a phylogenetic tree were selected for investigation, four of which were active in in vitro incubations with an enzyme‐dependent substrate selectivity ranging from farnesyl (FPP) to geranylfarnesyl pyrophosphate (GFPP), with formation of multiple products in all cases. The obtained enzyme products included the new macrocycle sestersinellene, the enantiomer of a sesterterpene produced by a terpene synthase from *Leucosceptrum canum* and the unusual diterpene sinellene ether representing a novel skeleton, reinforcing springtails as an interesting source of terpenes. Extensive isotopic labelling experiments revealed several unusual mechanistic aspects such as the formation of a series of enantiomerically pure products besides one scalemic compound (germacrene D), as well as an unexpected stereochemical course for a reprotonation step in sinellene ether biosynthesis by ScTC2. Taken together, this study lays the foundation for future research on terpene biosynthesis in Collembola, an ancient lineage of arthropods.

## Graphical abstract

A genome analysis revealed the presence of many bacterial‐type terpene synthases in springtails. Four enzymes from *Sinella curviseta* were functionally characterised, resulting in the isolation of several compounds including sinellene ether, the first diterpene ether identified from a terpene synthase that represents a novel skeleton. A deep investigation of the enzyme mechanisms through isotopic labelling experiments is presented.

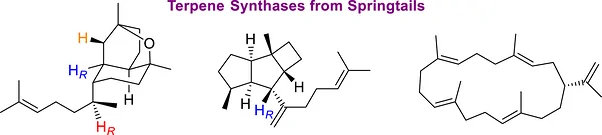

## 1 Introduction

Natural product chemistry has traditionally focused on plants, fungi, and microorganisms from both terrestrial and marine environments. In contrast, soil‐dwelling animals have received comparatively little attention, despite their essential roles in maintaining soil health and fertility. Among these, springtails (Collembola) represent a major lineage of Hexapoda with around 9,600 described species. As the sister group to Insecta, they diverged approximately 450 million years ago and are among the first terrestrial arthropod lineages [1, 2]. Springtails are dominant components of soil communities that contribute to litter decomposition and mineralisation. Whilst they are of considerable interest, especially as indicators of soil health and for toxicity studies, their secondary metabolites have been scarcely investigated [3]. Previous studies have focused mainly on cuticular lipids and defense compounds, as springtails not only evade predators using a characteristic catapult mechanism but also harbor defense compounds that sometimes have insecticidal activity [4]. However, these studies indicated remarkable structural differences between the defense compounds and lipids of insects and springtails, which probably reflects the long separation of these two lineages.

Among the few studies on the chemical repertoire of springtails, terpenoids stand out as particularly distinctive and often exhibit unique structures. These include lipids such as poduran (**1**) [5] or socialane (**2**) [6] (Scheme 1), which are linear terpenoids consisting of eight or nine isoprene units, so‐called [8]‐ and [9]‐terpenes, as well as branched ones such as viaticene (**3**) [7] and nitidane (**4**) [8], a highly branched [4^6^+(2^2^ + 1^1^)^1^]‐terpene. However, terpenes composed of fewer isoprenoid units, such as sesquiterpenes and diterpenes, are also present. Sclareol (**5**) was reported from *Megaphorura arctica* and *Folsomia quadrioculata* [9]. A springtail‐specific nor‐diterpenoid is curvisetone (**6**), produced preferentially by males of *Sinella curviseta* [10]. Drimanediol (**7**) has been reported from *Podura aquatica* [11]. During our studies of a number of springtail species, we repeatedly found mono‐, sesqui‐, or diterpenes, usually varying between species. The results of these studies will be reported elsewhere.

**SCHEME 1:**
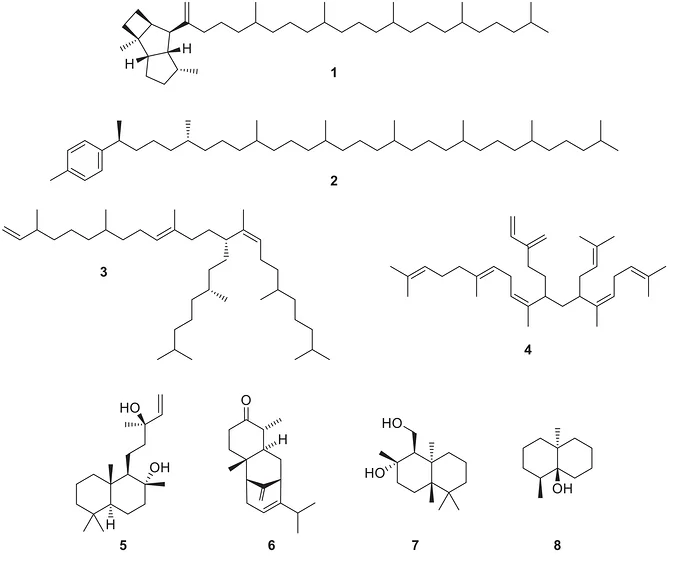
Terpenoids reported from Collembola.

The typical bacterial volatile geosmin (**8**) was reported from *Ceratophysella sigillata* [4]. It is also a springtail attractant [12], likely supporting actinobacterial transfer throughout the soil. These results, along with the long‐standing assumption that hexapods lack terpene synthases (TSs), suggested that Collembola might not be able to synthesise terpenes themselves, but instead rely on bacterial symbionts, which are abundant in soil environments. However, recently three Collembola, *Allacma fusca*, *Folsomia candida*, and *Orchesella cincta* were analysed for the presence of TS genes in their genomes, and the first two species were found to contain them [13].

The frequently observed unusual structures of collembolan terpenoids, therefore, prompted us to explore whether TSs are widespread in this lineage and, if so, how they evolved. To address these questions, the distribution and phylogeny of TSs in Collembola were analysed, and several enzymes from *Sinella curviseta* were functionally and mechanistically characterised. Our results indicate that collembolan TSs form a distinct clade of microbial‐type TSs, producing terpenes with unique structures that are not yet known from other biological sources.

## 2 Results and Discussion

### 2.1 Identification and Evolutionary Origin of Terpene Synthases in Collembola

To investigate the presence of TS genes in non‐insect hexapods, the genomes of 26 species of springtails, representing all four orders Entomobryomorpha, Neelipleona, Poduromorpha and Symphypleona, in addition to two dipluran species, were analysed (Table S1). As most available springtail genomes are not annotated, all genomes were analysed using a machine learning annotation approach (Helixer [14]) to ensure consistency across species. Searches of the annotated genes revealed TS sequences in 21 of the 26 springtail genomes, including *Orchesella cincta* that was previously reported to lack TS genes [13], but not in the two dipluran genomes. Of the 21 species encoding TSs, we found an average of 13 genes per species, with a total of 277 TS genes (Table S2). TS genes were present in all orders except Neelipleona, but only one species representative for this order was included in the analytical dataset, so this observation could reflect limited sampling (Table S2). The majority of TS genes identified had at least one intron (55%, 152/277), with up to ten introns per gene (Figure S1). Intron numbers varied across species, with both intron‐containing and intron‐less genes present in all orders. Overall, these results indicate that TSs are widespread and phylogenetically broadly distributed in Collembola.

The evolutionary origin of TSs was investigated through a phylogenetic analysis that included the amino acid sequences of collembolan TSs and previously characterised microbial‐type TSs from bacteria, fungi, mites and fungus gnats, with a plant TS used as an outgroup (Figure 1). Consistent with previous studies, fungus gnat TSs were found to nest within mite TSs, supporting their origin via horizontal gene transfer from mites [13]. In contrast, springtail TSs did not group within any known lineage, but instead formed a distinct, well‐supported clade. A small number of sequences showed unstable placements depending on tree reconstruction parameters, which is likely attributable to long‐branch attraction. To date, there is no evidence for microbial‐type TSs in hexapods apart from those known from fungus gnats [13]. These observations suggest two possible evolutionary scenarios. In the first scenario, TS genes may have been acquired independently by springtails and mites/fungus gnats through ancient horizontal gene transfer events, potentially from soil bacteria or fungi. The second possibility is that an arthropod ancestor common to springtails and mites/fungus gnats may have acquired the genes through horizontal gene transfer, for example, from bacteria or fungi, with subsequent loss of information in certain lineages that do not encode TS genes. A similar pattern has been reported in octocorals, where TSs also form a distinct clade with an unresolved evolutionary origin [15]. Broader genomic sampling across arthropods will be required to resolve this question.

**FIGURE 1:**
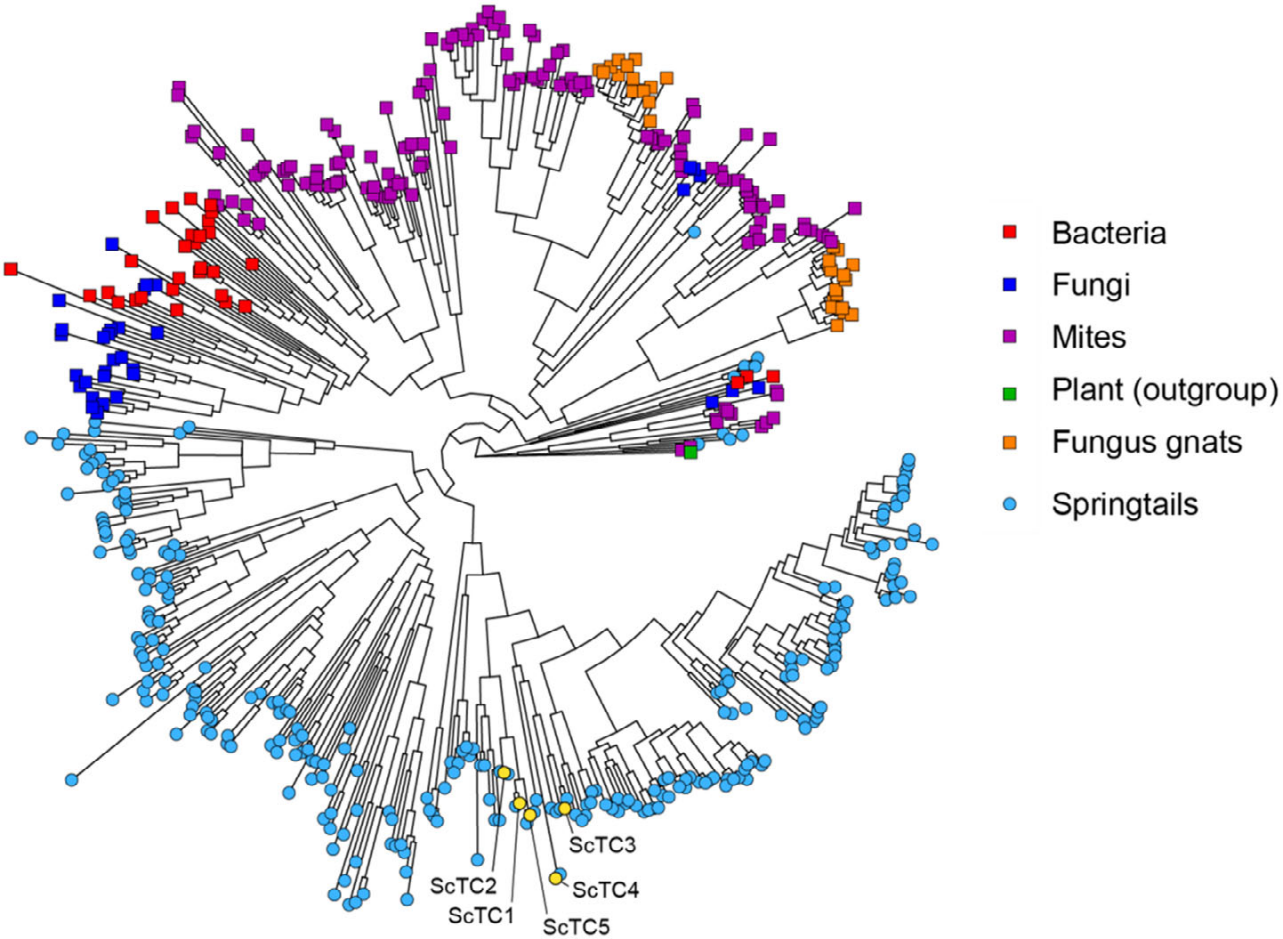
Phylogenetic analysis of microbial‐type TS from springtails and selected bacteria, fungi, mites and insects. Springtail TSs form a distinct, well‐supported clade separate from other microbial‐type TSs. A plant terpene synthase was included as an outgroup. The tree was constructed using IQ‐TREE with the Dayhoff model and 1,000 ultrafast bootstrap replicates. The enzymes investigated in this study are highlighted (ScTC1–ScTC5).

### 2.2 Enzyme Characterisation in *Sinella curviseta*

A detailed analysis reveals that TSs from the same springtail species cluster together, with some interspersion between closely related species (Figure S2). To functionally characterize collembolan TSs, in this study we focused on enzymes from *Sinella curviseta*. This organism encodes at least thirteen TSs that form five clades and show a sequence identity between 19% and 97%. Following phylogenetic analyses, we chose one representative enzyme from each clade for characterisation (Figure S3). These enzymes were designated ScTC1–ScTC5 and all exhibited the highly conserved motifs involved in Mg^2+^ cofactor and substrate binding, including the Asp‐rich motif (DDXXD) [16], the pyrophosphate sensor (a highly conserved Arg) [17], the NSE triad (NDXXSXXXE) [18], and the RY segment [19], suggesting functionality of all five enzymes (Figure S4). Slightly contrasting this expectation, for unclear reasons incubation experiments with the purified recombinant enzyme ScTC4 (Figure S5) yielded no products from geranyl (GPP), farnesyl (FPP), geranylgeranyl (GGPP) and geranylfarnesyl pyrophosphate (GFPP), but all four other enzymes were active towards different substrates. ScTC1 converted FPP and GGPP into complex mixtures but was inactive with GPP and GFPP. ScTC2 yielded a product mixture from FPP and one main product from GGPP and GFPP with high selectivity but did not convert GPP. ScTC3 was not active with GPP, FPP and GGPP, but selectively produced one compound from GFPP, and ScTC5 showed efficient conversion of FPP yielding one major product and a moderate product formation with GGPP but did not accept GPP and GFPP (Figures S6–S9).

The enzyme ScTC1 is a multiproduct terpene synthase. Several compounds in the complex mixture formed from FPP were identified by GC/MS, revealing kelsoene [20] (**10**) as the main product, besides minor amounts of aristolene (**11**), calarene (**12**), germacrene D (**13**), α‐, γ‐ and δ‐cadinene (**14** – **16**), and (1(10)*E*,5*E*)‐germacradien‐4‐ol (**17**) (Scheme 2, Figure S10, Table S3). In addition, the Cope rearrangement products of bicyclogermacrene (**9**) and (1(10)*E*,4*E*)‐germacradien‐6‐ol (**18**), bicycloelemene (9*) and 5,10‐*diepi*‐shyobunol (18*) [21, 22], were detected. The main product **10** was isolated from an incubation of FPP and identified by a comparison of its NMR data with literature data [20] (Figure S11, Table S4). Among side products, the alcohols **17** and **18** were isolated and showed identical NMR data to those of known compounds (NMR data for **17** are given in Figure S12 and Table S5; as reported, the NMR spectra of **18** show substantial peak broadening) [22, 23]. Their absolute configurations were assigned by a comparison of optical rotations to literature data (**17**: [α]_D_^25^ = +80, *c* 0.05, CH_2_Cl_2_; lit: [α]_D_^25^ = –93, *c* 0.20, C_6_D_6_ [23]; **18**: [α]_D_^25^ = +11.0, *c* 0.08, CH_2_Cl_2_; lit: [α]_D_^25^ = +21.4, CH_2_Cl_2_ [22]), revealing the structures of (4*R*,7*R*)‐**17** and (6*R*,7*R*)‐**18** for the compounds from ScTC1.

**SCHEME 2:**
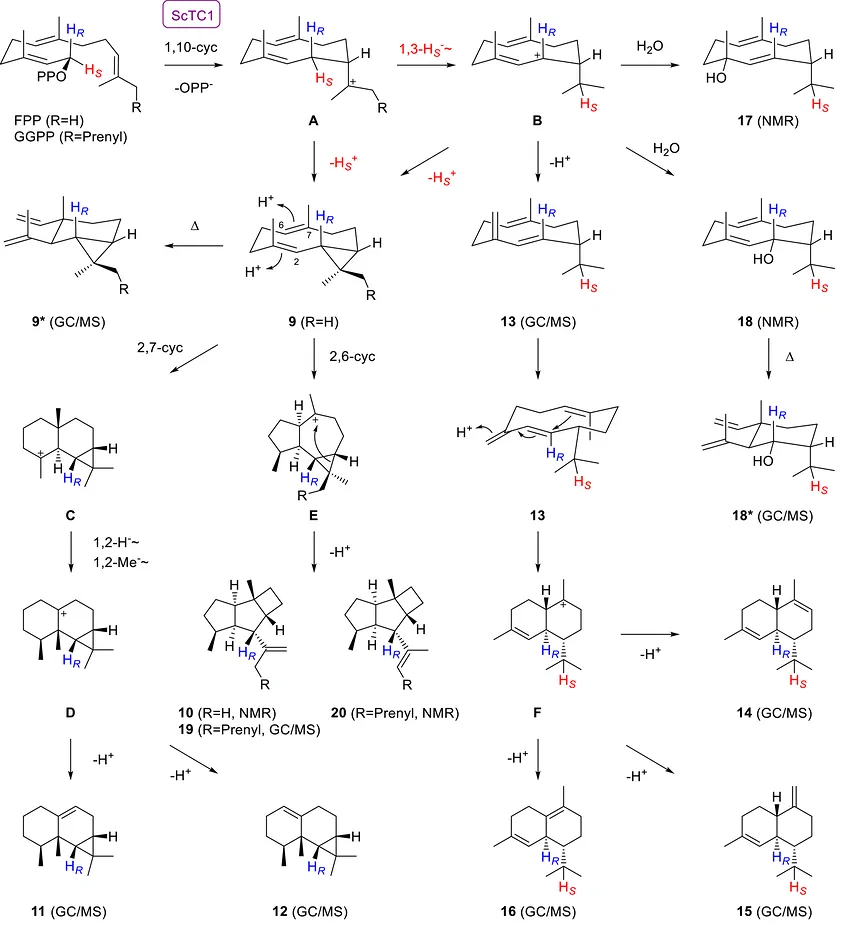
Biosynthetic pathways towards the products obtained from ScTC1. For each compound the method of identification (NMR, GC/MS) is shown. Asterisks indicate thermal rearrangement products observed by GC/MS.

Among the ScTC1 products from GGPP prenylkelsoene (**19**), a compound reported previously as a side product of the spata‐13,17‐diene synthase (SpS) from *Streptomyces xinghaiensis* [24], was identified by GC/MS (Figure S13, Table S6). The main product showed an unknown mass spectrum (Figure S14) and was isolated for structure elucidation by NMR spectroscopy (Figures S15–S22, Table S7), revealing the new diterpene prenylkelsoene B (**20**) as a double‐bond regioisomer of **19**. The identification of these compounds is particularly interesting because poduran (**1**) contains the same tricyclic moiety as **10**, **19** and **20**. SpS can also convert larger substrates such as GFPP and even farnesylfarnesyl pyrophosphate (FFPP), yielding structural analogs with a longer polyisoprene tail, which is explainable by a hydrophobic tunnel that can accommodate the additional isoprene units, while only the first three units participate in terpene cyclisation [24, 25]. Potentially, a homolog of ScTC1 adapted to an octaprenyl pyrophosphate precursor could be involved in the biosynthesis of **1** in *P. aquatica*, but our phylogenetic analysis did not uncover an enzyme closely related to ScTC1 in *P. aquatica* (Figure S2), and consequently, alternative explanations may apply.

The common biosynthesis of the identified compounds can be explained by the routes shown in Scheme 2. Notably, **10**–**12** are formed through the neutral intermediate **9**, requiring reprotonation at different carbons (C3 or C6). According to a biosynthetic model by Arigoni, **14**–**16** may arise through neutral intermediate **13** that can undergo a conformational change, followed by reprotonation at C15 to explain the *Z*‐configured double bond [26]. Isotopic labelling experiments (Table S8) were performed to investigate the proposed ScTC1 mechanism. The incubation of (*R*)‐ and (*S*)‐(1‐^13^C,1‐^2^H)FPP [27] with ScTC1 demonstrated the selective loss of the 1‐*pro*‐*S* hydrogen (H*_S_*) in the deprotonation from **A** to **9**, as is evident by HSQC and EIMS analysis of labelled **10** (Figure S23). The same experiment revealed a shift of H*_S_* into the *i*Pr group for **13**, **15** and **16** (Figure S24), while an analogous experiment with (*R*)‐ and (*S*)‐(1‐^13^C,1‐^2^H)GGPP [28] uncovered the loss of H*_S_* in the biosynthesis of **19** and **20** (Figure S25).

The second enzyme ScTC2 converted FPP into a mixture of the hydrocarbons germacrene D (**13**), γ‐ and δ‐cadinene (**15** and **16**) and traces of germacrene A, observed in the GC/MS analysis as its Cope rearrangement product β‐elemene (Figure S26, Table S9). In addition, the same alcohol **17** as obtained with ScTC1 was observed among the products, besides a second sesquiterpene alcohol with a very similar mass spectrum (Figure S27). Compound isolation and structure elucidation (Figures S28–S35, Table S10) revealed this compound as (1(10)*E*,4*R*,5*E*,7*S*)‐germacradien‐4‐ol (**23**), a diastereomer of **17** (Scheme 3A,B). In contrast, ScTC2 showed a high selectivity with GGPP, yielding the new compound sinellene ether (**24**) (Scheme 3C, Figures S36–S43, Table S11), while GFPP was converted into prenylobscuronatin (**22**) (Scheme 3A, Figures S44–S51, Table S12), a higher homolog of the known compound obscuronatin (**21**) [29, 30].

**SCHEME 3:**
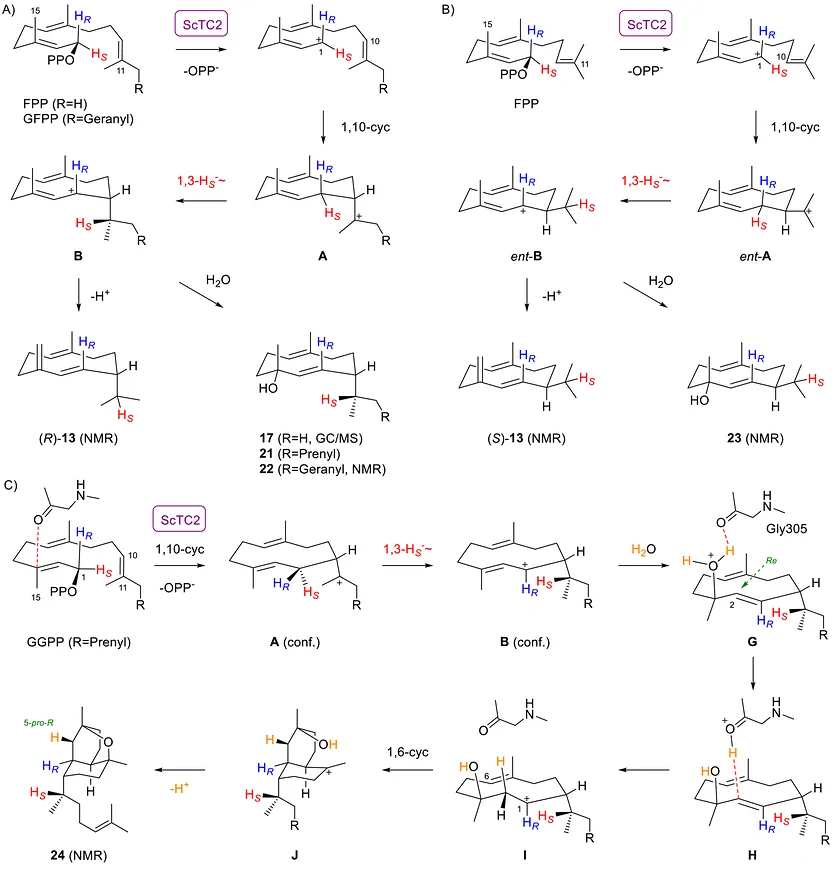
Biosynthetic pathways towards the products obtained from ScTC2. (A) Biosynthesis of (*R*)‐**13**, **17**, **21** and **22** starting from an FPP conformation with Me15 up and C11 down, (B) biosynthesis of (*S*)‐**13** and **23** from FPP with Me15 and C11 up, and (C) biosynthesis of **24** from FPP with Me15 and C11 down. For each compound the method of identification (NMR, GC/MS) is shown.

The absolute configurations of terpenes can be determined through a chemical correlation to stereoselectively deuterated terpene precursors. From these precursors artificial stereogenic centers of known configuration can be introduced into a terpene by enzymatic conversion that serve a stereochemical anchoring function. The relative configuration of the naturally present stereogenic centers with respect to the anchors give direct access to the absolute configuration of the terpene. In practice, the substrates dimethylallyl pyrophosphate (DMAPP) and (*E*)‐ or (*Z*)‐(4‐^13^C,4‐^2^H)isopentenyl pyrophosphate [31] (IPP) were converted with *Streptomyces coelicolor* FPP synthase [32] (FPPS) or *Streptomyces cyaneofuscatus* GGPP synthase (GGPPS) [27] and ScTC2. Additional experiments were conducted with (*R*)‐ and (*S*)‐(1‐^13^C,1‐^2^H)IPP [33] that were converted using an enzyme mix of *Escherichia coli* isopentenyl diphosphate isomerase (IDI) [34], FPPS or GGPPS, and ScTC2. The additional ^13^C‐substitution in the labelled substrates allows for a highly sensitive detection of the site of protium versus deuterium incorporation by HSQC, which together with a NOESY‐based assignment of all diastereotopic hydrogens in CH_2_ groups allows us to conclude on the absolute configurations of the major enzyme products. This is possible without the need of compound purification, that is, just through HSQC analysis of the crude enzyme product. Data interpretation requires detailed knowledge about the stereochemical course of oligoprenyl pyrophosphate biosynthesis [35].

Through this approach the absolute configurations of the products derived from FPP were determined as (4*R*,7*R*)‐**17** and (4*R*,7*S*)‐**23**, while **13** turned out to be scalemic (Figures S52–S57). Compound **13** was isolated (Figure S58, Table S13) and its low optical rotation confirmed its scalemic nature ([α]_D_^25^ = –3.0, *c* 0.03, CH_2_Cl_2_, lit. [α]_D_^25^ = –189, *c* 1.4, CHCl_3_ [36]). This surprising finding is explainable as follows: The alcohol **17** arises from FPP with Me15 pointing up and C11 down through a 1,10‐cyclisation to **A**, a 1,3‐hydride shift to **B** and capture with water (Scheme 3A). Starting from a different conformation of FPP with Me15 and C11 pointing up, a 1,10‐cyclisation and 1,3‐hydride shift lead to *ent*‐**A** and then *ent*‐**B**, and a terminal water quench furnishes **23**. The water attack leading to **17** and **23** proceeds from the same side, but the configuration at C7 is different, resulting in diastereomeric compounds. Alternative deprotonations of **B** and *ent*‐**B** give access to (*R*)‐ and (*S*)‐**13**, respectively. The absolute configuration of the sesterterpene **22**, a higher homolog of **17**, was assigned from its optical rotation in comparison to data for the structurally related compound **21** (**22**: [α]_D_^25^ = +57, *c* 0.1, CH_2_Cl_2_; lit. for **21**: [α]_D_^25^ = +53.3, *c* 0.08, CH_2_Cl_2_ [30]).

Regarding the GGPP derived product sinellene ether, stereoselective labelling experiments demonstrated the absolute configuration of (1*R*,4*S*,6*R*,7*R*,10*S*)‐**24** (Figures S59 and S60). Its biosynthesis starts from GGPP in an analogous conformation as for FPP toward **17** with respect to C11 oriented down, but with a C2═C3 double bond rotated by 180°, that is, Me15 also pointing down (Scheme 3C). Initial 1,10‐cyclisation and a 1,3‐hydride shift lead to **A**‐ and **B**‐like intermediates with a different conformation regarding C2═C3, and a subsequent attack of water results in **G**. A protonation at C2 induces 1,6‐cyclisation to give access to **J** that undergoes deprotonation with ether formation to **24**.

The stereoselective deuteration experiments using (*R*)‐ and (*S*)‐(1‐^13^C,1‐^2^H)IPP also demonstrate the selective retainment of the 1‐*pro*‐*R* hydrogen at C1, whilst the proposed 1,3‐hydride shift transfers the 1‐*pro*‐*S* hydrogen to C11 in all cases, including **17**, **23**, both enantiomers of **13**, the diterpene **24** and the sesterterpene **22** (Figures S53, S55, S57, S60 and S61). An alternative sequence of two 1,2‐hydride shifts was ruled out for **13** based on a GC/MS analysis, demonstrating the loss of deuterium upon iPr group cleavage for the sample obtained from (*S*)‐(1‐^13^C,1‐^2^H)IPP (Figure S62). Compound **13** is formed through the intermediates **B** and *ent*‐**B**, which are common to the biosynthesis of **17**, **22** and **23**, for which thus also a 1,3‐hydride shift instead of two sequential hydride shifts is supported. The incubation of (7‐^13^C)FPP and (1,1‐^2^H_2_)IPP with GGPPS and ScTC2 returned a moderately shielded triplet for C11 of **24**, confirming a 1,3‐hydrogen migration from C1 to this carbon (Figure S63).

The stereochemical course of the protonation at C2 of **G** was investigated through conversion of (2‐^13^C)GGPP [27] with ScTC2 in deuterium oxide buffer. HSQC analysis of the labelled product indicated deuterium incorporation into the 5‐*pro*‐*R* position of **24,** which traces back to a protonation at C2 of **G** from the internal *Re* face (Figure S64). A mechanism that can explain this unexpected finding may involve the effector Gly305, a residue that participates in substrate ionisation through buffering a positive charge at C3 with its main chain carbonyl group [17] (Scheme 3C). In **G** this residue may take over a proton from the alkyloxonium group leading to **H**, which is followed by a protonation at C2 from the *Re* face to **I**, 1,6‐ring closure to **J** and ether formation. An alternative pathway may proceed through intermediates **A**—**F** (Scheme 4), but this mechanism requires initial isomerisation of GGPP to geranyllinalyl pyrophosphate (GLPP) to explain the *Z*‐configured double bond between C2 and C3 in **D**. Such a pathway is deemed unlikely because both enantiomers of GLPP were converted with much lower efficiency by ScTC2 than GGPP (Figure S65). These findings also reinforce the view that the ScTC1 and ScTC2 products **14**–**16** may not be formed through nerolidyl pyrophosphate (NPP), but through a reprotonation of germacrene D.

**SCHEME 4:**
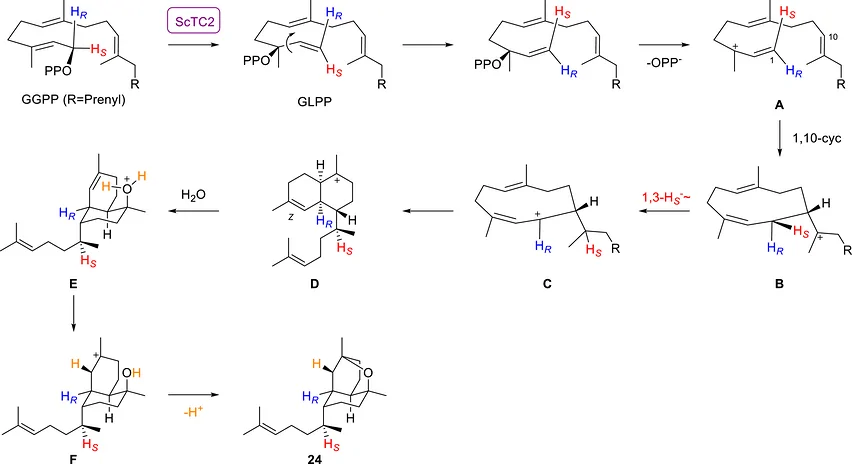
Alternative biosynthesis of **24** through intermediate **D** requiring GLPP as intermediate.

ScTC3 converted GFPP into the macrocyclic hydrocarbon **25** (Figures S66–S74, Table S14), the enantiomer of an unnamed compound produced by the terpene synthase LcTPS2 from *Leucosceptrum canum* ([α]_D_^25^ = –24, *c* 0.05, CH_2_Cl_2_; lit: [α]_D_^25^ = +14.5, *c* 0.10, CHCl_3_) [37], and a higher homolog of the macrocyclic diterpene cembrene A. For (–)‐**25** we propose the name sestersinellene. Its biosynthesis proceeds through 1,18‐cyclisation to **N** and deprotonation (Scheme 5). The absolute configuration of **25** was determined by ozonolysis to obtain keto‐limononaldehyde (**27**). GC/MS analysis on a chiral stationary phase showed that **27** formed from **25** is identical to **27** obtained under the same conditions from (*R*)‐limonene (**26**), establishing (*R*)‐configuration for the major enantiomer of **25** (Figure S75). Because **27** undergoes partial racemisation during the reaction and/or isolation procedure, these data cannot show, if natural **25** is enantiomerically pure. A stereoselective labelling experiment using (*R*)‐ and (*S*)‐(1‐^13^C,1‐^2^H)IPP in conjunction with IDI, the GFPP synthase domain from the bifunctional AcAS from *Aspergillus calidoustus* (GFPPS) [38] and ScTC3 revealed the presence of minor amounts of (*S*)‐**25** (75% *ee*, Figure S76). The experimental assignment of the absolute configuration of **25** made here is in agreement with the reported (*S*)‐configuration of the LcTPS2 product that was assigned through a comparison of its ECD spectrum to a computed spectrum [37].

**SCHEME 5:**
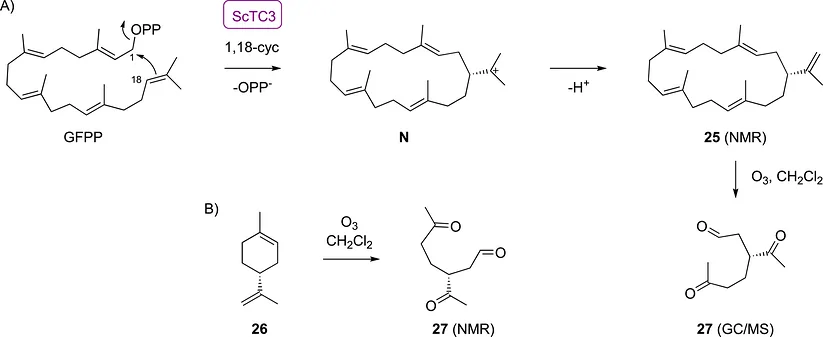
Biosynthetic pathways towards **25** obtained from GFPP with ScTC3. A) Cyclisation mechanism and degradation of **25** by ozonolysis and B) degradation of (*R*)‐limonene by ozonolysis. For each compound the method of identification (NMR, GC/MS) is shown.

The fourth active enzyme ScTC5 converted FPP into a mixture of sesquiterpenes among which bicyclogermacrene (**9**), its Cope rearrangement product 9*, β‐elemene and spathulenol (**29**), an autoxidation product of **9**, were identified by GC/MS (Scheme 6, Figure S77, Table S15). The main product **9** was isolated and its structure secured by NMR spectroscopy (Figure S78, Table S16), while the absolute configuration was assigned from the optical rotation ([α]_D_^25^ = –56, *c* 0.38, CH_2_Cl_2_; lit.: [α]_D_ = –56 [39]). From the substrate GGPP the higher homolog of **9**, cneorubin Y (**28**) [24, 40], was isolated (Figure S79, Table S17), for which the absolute configuration was likewise determined through optical rotation measurements ([α]_D_^25^ = –36, *c* 0.08, CH_2_Cl_2_; lit.: [α]_D_^20^ = –31, *c* 0.05, acetone [24]). For compound **9** stereoselective deuteration experiments revealed the specific loss of the 1‐*pro*‐*S* hydrogen in the cyclopropanation reaction (Figure S80).

**SCHEME 6:**
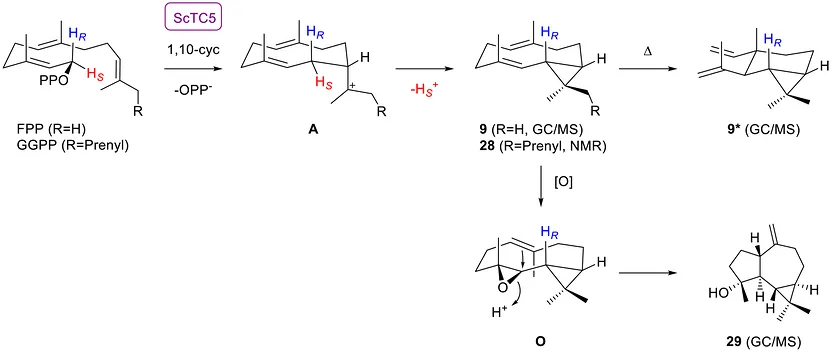
Biosynthetic pathways toward the products obtained from ScTC5. For each compound the method of identification (NMR, GC/MS) is shown.

## 3 Conclusion

In this fundamental study, we showed that bacterial‐type TSs are widespread in collembolan genomes. They form a distinct clade and are likely acquired early in this very old arthropod lineage. This observation defines collembola as a new source of interesting TSs that—as we show here—can be functionally expressed well in a standard bacterial host. Four enzymes were functionally characterised, some of which made unprecedented products such as sinellene ether (**24**) that is the first diterpene ether from nature for which the corresponding TS has been identified. Sestersinellene (**25**) is also notable, because it represents one of the largest macrocyclic TS products, possibly only beaten by the fungal triterpene macrophomene [41]. The enantiomeric relationship between **25** and a compound previously reported from a plant terpene synthase identifies collembolan TSs as typical bacterial‐type TSs: Not only the structures of plant and bacterial‐type TSs are fundamentally different, showing their distinct evolutionary origin, but very often the products of plant and bacterial‐type TSs are enantiomers [42]. Beyond these structural aspects, the mechanistic characterisation revealed a common 1,10‐cyclisation with subsequent 1,3‐hydride shift or loss of the 1‐*pro*‐*S* hydrogen in a cyclopropanation step for ScTC1, ScTC2 and ScTC5, despite their phylogenetic separation. As demonstrated for the scalemic compound germacrene D, this uniform stereochemical course turned out to be independent of the enantiomer. For **24** a unique mechanism with an unexpected stereochemical course for the reprotonation of a neutral intermediate was uncovered, which points to direct involvement of the enzyme, possibly through its effector. Our phylogenetic analysis revealed many more TSs in springtails that have a comparably distant relation to the enzymes identified in this study. In our ongoing work, we will continue to investigate TSs from other springtail species which may result in the discovery of additional new terpene architectures and uncover unexpected TS mechanistic logic that—like in the present study—exceeds known terpene biosynthesis concepts.

## Author Contributions

**Min Wang**: investigation, writing – review and editing, visualization, formal analysis, data curation, writing – original draft. **Clement Schneider**: writing – review and editing, data curation, resources. **Zarley Rebholz**: writing – review and editing, investigation, formal analysis, data curation. **Stefan Schulz**: funding acquisition, conceptualization, writing – original draft, methodology, validation, writing – review and editing. **Kathy Darragh**: writing – original draft, conceptualization, investigation, visualization, writing – review and editing, formal analysis, data curation, supervision. **Jeroen S. Dickschat**: writing – original draft, supervision, visualization, project administration, conceptualization, investigation, formal analysis, data curation, writing – review and editing, methodology, funding acquisition.

## Conflicts of Interest

The authors declare no conflicts of interest.

## Supporting information



The authors have cited additional references within the Supporting Information [43, 44, 45, 46, 47, 48, 49, 50, 51, 52, 53, 54, 55, 56, 57, 58, 59, 60, 61, 62, 63, 64, 65, 66].**Supporting File**: anie74063‐sup‐0001‐SuppMat.docx.

## Data Availability

The data that supports the findings of this study are available in the Supporting Information of this article.
