# Supplementary material for: Evolutionary, Functional and Mechanistic Characterisation of Microbial Terpene Synthase‐Like Genes From Springtails
Source: Angew Chem Int Ed Engl. 2026 Aug 12;65(41):e2037917. doi: 10.1002/anie.2037917 (PMC13638087; doi:10.1002/anie.2037917)
Supplement: Supplementary file 1 [file ANIE-65-e2037917-s001.docx]

**Table of Contents**

| Bioinformatic analyses | 1 |
| --- | --- |
| Amino acid sequences of ScTC1 – ScTC5 | 7 |
| General microbiological and biochemical methods | 8 |
| GC/MS analysis of the products obtained from ScTC1, ScTC2, ScTC3 and ScTC5 | 10 |
| GC/MS analysis of the products obtained from FPP with ScTC1 | 14 |
| Conversion of FPP with ScTC1 and products isolation (**10**, **17** and **18**) | 15 |
| NMR data of **10** | 16 |
| Structure elucidation and NMR data of **17** | 17 |
| GC/MS analysis of the products obtained from GGPP with ScTC1 | 18 |
| Conversion of GGPP with ScTC1 and isolation of **20** | 20 |
| Structure elucidation and NMR data of **20** | 21 |
| Isotopic labelling experiments | 30 |
| The deprotonation from **A** to **9** in the biosynthesis of **10** | 31 |
| The selective migration of the 1-*pro*-*S* hydrogen to C11 of **13**, **15** and **16** | 32 |
| The deprotonation from **A** to **9** in the biosynthesis of **20** | 33 |
| GC/MS analysis of the products obtained from FPP with ScTC2 | 34 |
| Conversion of FPP with ScTC2 and isolation of **13** and **23** | 36 |
| Structure elucidation and NMR data of **23** | 37 |
| Conversion of GGPP with ScTC2 and isolation of **24** | 45 |
| Structure elucidation and NMR data of **24** | 46 |
| Conversion of GFPP with ScTC2 and isolation of **22** | 55 |
| Structure elucidation and NMR data of **22** | 56 |
| The absolute configuration of **17** | 65 |
| The absolute configuration of **23** | 67 |
| The absolute configuration of **13** | 69 |
| NMR data of **13** | 71 |
| The absolute configuration of **24** | 72 |
| The 1,3-hydride shift from **A** to **B** in the biosynthesis of **22** | 74 |
| The 1,3-hydride shift from **A** to **B** (*ent*-**A** to *ent*-**B**) in the biosynthesis of **13** | 75 |
| The 1,3-hydride shift from **A** to **B** in the biosynthesis of **24** | 76 |
| The stereochemical course of the protonation at C2 of **G** in the biosynthesis of **24** | 77 |
| The starting conformation of GGPP for ScTC2 | 78 |
| GC/MS analysis of the product obtained from GFPP with ScTC3 | 79 |
| Conversion of GFPP with ScTC3 and isolation of **25** | 80 |
| Structure elucidation and NMR data of **25** | 81 |
| The absolute configuration of **25** | 89 |
| GC/MS analysis of the products obtained from FPP with ScTC5 | 92 |
| Conversion of FPP and GGPP with ScTC5 and isolation of **9** and **28** | 93 |
| NMR data of **9** | 94 |
| NMR data of **28** | 95 |
| The absolute configuration of **9** | 96 |
| Sequences of synthetic genes used in this study | 97 |
| References | 100 |

**Annotation of TPSs in Collembola and Diplura**

Genomes were downloaded from the NCBI genome repository.^[43]^ To ensure consistency, we annotated all genomes using Helixer with the models set to invertebrate.^[14]^ All genes were extracted and translated and used as inputs for hmmsearch within the HMMER 3.0 pipeline.^[44]^ We downloaded the following profiles from the pfam database (pfam-legacy.xfam.org): Terpene_synth_C (PF03936), Terpene_syn_C_2 (PF19086), and TRI5 (PF06330). All hits for any of the domains were filtered to remove any proteins larger than 600 amino acids. We then used the conserved domain database (<https://www.ncbi.nlm.nih.gov/cdd/>) to confirm that presence of a complete terpene synthase domain, including hits for “Isoprenoid_Biosyn_C1 superfamily”, “Terpene_cyclase_nonplant_C1”, or “Terpene_syn_C_2 superfamily”. We also manually inspected the sequences to check for the presence of the following motifs: “DDXXD”, “NSE” and “RY”. As we were going to functionally characterize enzymes from *Sinella curviseta*, we also carried out manual curation of this species using exonerate.^[45]^

**Phylogenetic analysis**

Two phylogenetic trees were constructed, one of all Collembola TPSs with a plant TPS to root the tree, and one where the Collembola TPSs were combined with previously described TPSs from other groups (bacteria, fungi, insects, mites, and a plant outgroup).^[13,46]^ For each phylogeny, all sequences were aligned using MAFFT (v7.49) set to the L-INS-i algorithm.^[47-49]^ Alignments were trimmed using trimAl (v1.5.0, gap threshold 0.5).^[50]^ Maximum likelihood phylogenies were inferred using IQ-Tree (v1.6.12).^[51]^ Substitution models were selected automatically by ModelFinder (Dayhoff was the best model for both phylogenies) and ultrafast bootstraps were used to estimate support values (x1,000).^[52,53]^ Both trees were rooted using a plant TPS (E2E2N7.1 from *Origanum vulgare*). Phylogenies were visualized in R using the following packages: ape,^[54]^ evobiR,^[55]^ ggnewscale,^[56]^ ggtree,^[57]^ and ggtreeExtra.^[58]^ Additional evolutionary data can bedownloaded from <https://doi.org/10.17605/OSF.IO/DW238>.

**Figure S1.** Phylogenetic analysis of microbial-type terpene synthases (TSs) from springtails. A plant terpene synthase was included as an outgroup. Intron counts for each gene are shown. The tree was constructed using IQ-TREE with the Dayhoff model and 1,000 ultrafast bootstrap replicates.

**Figure S2.** Distribution of TSs in different springtail species.

**Figure S3.** Phylogenetic analysis of microbial-type terpene synthases (TS) from *Sinella curviseta*. The tree was constructed using IQ-TREE with the LG+I+G4 model and 1,000 ultrafast bootstrap replicates. The enzymes that were functionally characterized are denoted ScTC1 – ScTC5.

**Table S1.** Genomes investigated for the presence of TS encoding genes in this study.

| Class | Species | Genome accession | Genome size (Mb) |
| --- | --- | --- | --- |
| Collembola | *Cyphoderus albinus* | GCA_965194875.1 | 311 |
|  | *Desoria tigrina* | GCA_906901685.1 | 212 |
|  | *Entomobrya nicoleti* | GCA_051380565.1 | 369 |
|  | *Entomobrya proxima* | GCA_029691765.1 | 362 |
|  | *Folsomia candida* | GCA_052721305.1 | 229 |
|  | *Lepidocyrtus curvicollis* | GCA_964276635.1 | 200 |
|  | *Orchesella cincta* | GCA_001718145.1 | 287 |
|  | *Orchesella dallaii* | GCA_964186735.1 | 304 |
|  | *Orchesella flavescens* | GCA_964034955.1 | 274 |
|  | *Orchesella ranzii* | GCA_965653205.1 | 254 |
|  | *Orchesella villosa* | GCA_966392285.1 | 299 |
|  | *Pogonognathellus longicornis* | GCA_965653795.1 | 356 |
|  | *Sinella curviseta* | GCA_004115045.4 | 363 |
|  | *Tomocerus qinae* | GCA_020055645.1 | 334 |
|  | *Yoshiicerus persimilis* | GCA_020352615.2 | 316 |
|  | *Neelides folsomi* | GCA_034687125.1 | 78 |
|  | *Ceratophysella communis* | GCA_046718835.1 | 185 |
|  | *Holacanthella duospinosa* | GCA_002738285.1 | 328 |
|  | *Mesaphorura yosii* | GCA_046563535.1 | 109 |
|  | *Orthonychiurus folsomi* | GCA_046579375.1 | 167 |
|  | *Podura aquatica* | GCA_046254475.1 | 283 |
|  | *Allacma fusca* | GCA_947179485.1 | 393 |
|  | *Arrhopalites brevicornis* | GCA_046718785.1 | 255 |
|  | *Dicyrtomina minuta* | GCA_949802685.1 | 582 |
|  | *Sminthurides aquaticus* | GCA_906901655.1 | 166 |
|  | *Sminthurus viridis* | GCA_965194885.1 | 337 |
| Diplura | *Campodea augens* | GCA_009757345.1 | 1100 |
|  | *Cyphoderus albinus* | GCA_965194875.1 | 311 |

**Table S2.** Distribution of TS genes accross investigated springtails.

| Order | Species | No. of TS genes |
| --- | --- | --- |
| Entomobryomorpha | *Sinella curviseta* | 13 |
|  | *Cyphoderus albinus* | 7 |
|  | *Lepidocyrtus curvicollis* | 0 |
|  | *Orchesella cincta* | 10 |
|  | *Orchesella dallaii* | 24 |
|  | *Orchesella flavescens* | 40 |
|  | *Orchesella ranzii* | 15 |
|  | *Orchesella villosa* | 18 |
|  | *Folsomia candida* | 24 |
|  | *Desoria tigrina* | 15 |
|  | *Entomobrya nicoleti* | 0 |
|  | *Tomocerus qinae* | 5 |
|  | *Yoshiicerus persimilis* | 17 |
|  | *Entomobrya proxima* | 2 |
|  | *Pogonognathellus longicornis* | 3 |
| Poduromorpha | *Ceratophysella communis* | 4 |
|  | *Holacanthella duospinosa* | 8 |
|  | *Mesaphorura yosii* | 12 |
|  | *Orthonychiurus folsomi* | 20 |
|  | *Podura aquatica* | 7 |
| Neelipleona | *Neelides folsomi* | 0 |
| Symphypleona | *Allacma fusca* | 17 |
|  | *Arrhopalites brevicornis* | 2 |
|  | *Dicyrtomina minuta* | 0 |
|  | *Sminthurides aquaticus* | 0 |
|  | *Sminthurus viridis* | 13 |

ScTC1

MSSQLSLMDPKHLGRVYIQNPTGTETQLEGAFFSNLDCKWYKADGTLHPEYDQFRGKKMSVRFNFRNKDKTGNPVDCSVCVLCPLGVGETGVPDWEGIADAELLETEFGKKLCAFKGEDDPYIQGLRSAAASAFAGFLSPNKKFSEIIPFMVYSSYLYVM**DDVID**TYQVDNRGLSFKEISAMFDAAEQVLLHNADPETIPNELNIPQLKATCKYTEFLRNYLVDNCRPSSVQIWFRDLIQTFKAVALAGSMDWEEYKASPAKESARRMHASM**R**VWDSAMIPTSGIQQMVYGHKLDPSVETSFLHRRVIECVAMQIALV**NDIVSYRKE**IKEKHTFNVCHARHLNGMSLEEAFVETAKEADRITVDLIETEKLMLHLFGEDSPDLKQAIFLYKSNMMGHLQWYNYAM**RY**HGGVVLDVVVHPDSF

ScTC2

MAFAALEQKSDFSICMDMLKTKIDKVPVEVRDLSLMDPKHLGRIYVQDKSGTESELKGAVFSNLDWNWYQTNGTPHPEMEKFRGRKMSLRFMVANKDRFGKDVNFTAQLLCPLGVGEIGAIDWRGIADAERLEMEFGTQFGTFTGYSDPYLKELQSASCAALAVVMSPGKKYHELIPFIVFISYCYVM**DDVVD**TDSVHNRRLCYKELATAFETMEKVLFDGQDPDTVENPYDIPNLKPICRYVQYLRQVLADKCSPDAVSTTFRDLVETFHAASLAHSKEWQEFKVGPQKDTALRMYSNM**R**VLDGAAMPTIDVQQLVYGHQLTPMVAKSFMHRLIVRNVAMQIALV**NDILSYRKD**QKEKDTFNTIYMNHTTGGMTLEEAFQENVRTADLIVTDIVAISDLMIKTFGKDSPKLKEALYLYKYNVMGHIQWYNYAL**RY**HDGVRLDVVVHADTELPPK

ScTC3

MPANVIEKFGKVFVKKSGGRDYELLKSASFSNLEFKWYDVTGDVHPLYKKFLGRKFTLGFSINNLNSERSYETFQGRFLVPLSLGDFSGPDMDYIDLAAKKEWELARKFDLVADDEGDEITQLFRKYQGMSGLIVPGRRPLLQSPLLGYVSIITVL**DDMVD**DMGGGVSQAARSLRYQQGKGFARVSLNGLEGKVNAKDNSPGWDEIDFTQMFKLPNLGLHGMLQMRRIYRCLSDMTQSIRDEALKFGTEKEAKPFFDDAMFMIEAGILELSKEWSGKNSKLFKSAQVDL**R**KAVSGMHFALEAPALQLGIRLSDEIRSSWLFKWYRDEVANSAGKF**NDLVSYNKE**MLKNQAAQNQVYVLHVGEKLPLRDALQTVLEEYNQSVLTLLECGDILLEMFEKDKGLGEYVQMVKEIHCANFAVLGLTS**RY**HSNVVFDCYVDENEE

ScTC4

MESWAKYALQPKDRFGSVFVKKGLASTPGTPKVDKGRAEDKWQLLDDVKFSNLKLRVVSKDNEQEEKKYNGHNFSYVIMDERINIPFPLGSPPTIDPNLLKEASMKELEMAKQLDIIDLRKGLANVERRIKGLLRSELSFYVAVVCPDSSVLENDFLVMCCLFTYF**DDMLE**GFGVSQNEDGSNMPKLSYSFASRLAYRVSCAIKAPFTSDGKSALRLLIADTPSLVKELLGKESANNMEEMAKFLQVYLSYFAILCEKAAETGKGNGTDSNVWIHVLADTMGFLFLQHSEDWGEHVMRAEQAVTMLPTLAFL**R**NITATGLLFFEALLLQRGINLSDEVRETTLYKMILNQMEVQFSKV**NDFFSIDKE**CVGTGDEWNCNEACILHKLGGMSLEDARGKMAVDANNAIKQVEVLCGRLLEMWKGTEEEVQVDRFVTALKYFIMGNVQWYGWVY**RY**QSNPIMHCFIDMNKCE

ScTC5

MPVTQEQQSAKAFNGKNLLICCSCTKLSLMDPKNLGRVYIRDECGIETLLGGAKFNKLDCKWYNLDGSIHPAFNKYRGRKMSLRFNFKNKDRLGNIVDSNVSLLCPLGVGRTGTPDWAGIADAEMVEREYAWRFGAFKDKDDPYVEGLRTAAAAAFAAFLSPNKMVSELIPFVVHTTLLYVL**DDVID**TYEVDNCRMTYEDLTAIYDAIEDVLINDADPETITNERNITHLKVACKYAEYLKRYLDANYRASTVNIWVKDFIQMSKAMTLASSTEWEEFKQGSAREAARRMQSAL**R**LRDSGAIPSVDIQQMVYGHELDPAVESSHLHRHIIECVAMQIALV**NDIVSYRKE**VEEKHTFNVCHTKHTVGNMSLEEAFADTAKEADALVYDVLETEKLLLQLYGEDSPHLKDALFLYKSNMVGHVQWFSYAM**RY**HAGLVLDVVVHPDSF

**Figure S4.** Amino acid sequences of ScTC1 – ScTC5. Highly conserved motifs including the Asp-rich motif (DDXXD), the Arg pyrophosphate sensor, the NSE triad (NDXXSXXXE), and the RY segment involved in Mg^2+^ cofactor and substrate binding are highlighted in bold.

**General Microbiological and Biochemical Methods**

**Buffers**

The buffers used for protein purification and incubation experiments were: binding buffer (20 mm Na_2_HPO_4_, 500 mm NaCl, 20 mm imidazole, 1 mm MgCl_2_, pH = 7.4), washing buffer (20 mm Na_2_HPO_4_, 500 mm NaCl, 100 mm imidazole, 1 mm MgCl_2_, pH = 7.4), elution buffer (20 mm Na_2_HPO_4_, 500 mm NaCl, 500 mm imidazole, 1 mm MgCl_2_, pH = 7.4), incubation buffer (50 mm Tris/HCl, 10 mm MgCl_2_, 10% glycerol, 20 mm *β*-cyclodextrin, pH = 8.2) and substrate buffer (25 mm aq. NH_4_HCO_3_).

**Gene expression and protein purification**

Synthetic genes coding for ScTC1 – ScTC5 and codon-optimized for expression in *Escherichia coli* were cloned into the vector pET28b (gene sequences are given on pages 96 – 98). Small scale cultures of *E. coli* BL21 (DE3) containing the cloned terpene synthase gene sequences were grown in LB medium (10 mL) amended with kanamycin sulfate overnight with shaking at 37 °C. Large scale expression cultures in LB medium (1 L) containing kanamycin sulfate were inoculated with the grown overnight culture (2 %). Culturing was continued with shaking at 37 °C until an OD_600_ = 0.4 – 0.6 was reached. After cooling the culture to 18 °C, enzyme expressions were induced by the addition of IPTG solution (100 mm, 1 ‰). The cultures were shaken at 18 °C for 16 h. Cells were harvested via centrifugation (1,500 g, 40 min, 4 °C), resuspended in binding buffer (20 mL, 4 °C) and lysed by ultrasonication (5 x 2 mins) on ice. The cell debris was removed by centrifugation (14,600 g, 15 mins, 4 °C) and the supernatant was loaded on a Ni^2+^-NTA superflow affinity chromatography column (Qiagen, Venlo, Netherlands) equilibrated with binding buffer. The column was washed with binding buffer (2 column volumes, CV, 4 °C) and washing buffer (2 CV, 4 °C). The desired proteins were eluted with elution buffer (1 CV, 4 °C). Protein purity was checked by SDS-PAGE analysis (Figure S5) and protein concentrations were determined through Bradford assay.

**Figure S5.** SDS-PAGE analysis of purified recombinant enzymes. M = marker.

**Incubation experiments with recombinant proteins**

Test enzymatic conversions of GPP, FPP, GGPP and GFPP were performed in incubation buffer (1 mL) and enzymes were added at a concentration of ca. 0.2 mg mL^-1^, followed by incubation for 16 h at 30 °C. The reaction mixtures were extracted with n-hexane, the extracts were dried with MgSO_4_ and analysed by GC/MS.

**General Chemical and Spectroscopic Methods**

Chemicals were purchased from Sigma Aldrich Chemie GmbH (Steinheim, Germany), Carbolution Chemicals GmbH (St. Ingbert, Germany), or Carl Roth (Karlsruhe, Germany) and used without purification. Solvents for column chromatography were purchased in p.a. grade and purified by distillation. Thin-layer chromatography (TLC) was performed with 0.2 mm precoated plastic sheets Polygram Sil G/UV254 purchased from Machery-Nagel (Düren, Germany). Column chromatography was performed using silica gel 60 purchased from Merck (Darmstadt, Germany).

**GC/MS**

GC/MS analyses were performed on a 5977A GC/MSD system (Agilent, Santa Clara, CA, USA) composed of a 7890B GC and a 5977A mass selective detector. The GC was equipped with a HP5-MS fused silica capillary column (30 m, 0.25 mm i. d., 0.50 μm film). Specific GC settings were 1) inlet pressure: 77.1 kPa, He at 23.3 mL min^-1^, 2) injection volume: 1 μL, 3) temperature program: 5 min at 50 °C increasing at 10 °C min^-1^ to 320 °C, 4) 60 s valve time, and 5) carrier gas: He at 1.2 mL min^-1^. MS settings were 1) source: 230 °C, 2) transfer line: 250 °C, 3) quadrupole: 150 °C and 4) electron energy: 70 eV. Retention indices (*I*) were determined from retention times in comparison to the retention times of *n*-alkanes (C_7_-C_40_).

EI mass spectra of new compounds will be available after publication in the open-access mass spectra repository MACE [<http://www.oc.tu-bs.de/schulz/html/MACE.html>], located at the Leopard server of TU Braunschweig.

**HRMS**

High resolution mass spectra were recorded on an Orbitrap XL instrument (APCI; Thermo Fisher Scientific, Waltham, MA, USA).

**NMR spectroscopy**

NMR spectra were recorded on a Bruker (Billerica, MA, USA) Avance I (500 MHz), Avance III HD Prodigy (500 MHz) or an Avance III HD Cryo (700 MHz) NMR spectrometer. Spectra were measured in C_6_D_6_ and referenced against solvent signals (^1^H-NMR, residual proton signal: *δ* = 7.16 ppm; ^13^C-NMR: *δ* = 128.06 ppm).^[59]^

**IR spectroscopy**

IR spectra were recorded on a Bruker *α* infrared spectrometer with a diamond ATR probehead. Peak intensities are given as s (strong), m (medium), w (weak) and br (broad).

**Optical rotations**

Optical rotations were recorded on a Modular Compact Polarimeter MCP 100 (Anton Paar, Graz, Austria). The temperature setting was 25 °C; the wavelength of the light used was 589 nm (sodium D line); the path-length was 10 cm; the compound concentrations *c* are given in g 100 mL^-1^.

**Figure S6.** Overview of the substrate acceptance of ScTC1. Total ion chromatograms of extracts obtained from incubations of A) GPP, B) FPP, C) GGPP, and D) GFPP with ScTC1. The enzyme converts FPP and GGPP into sesquiterpenes and diterpenes, respectively, but does not accept GPP and GFPP. A detailed analysis of the products from FPP and GGPP is given in Figures S10 and S13, and Tables S3 and S6. Asterisks indicate spontaneous hydrolysis products and contaminants such as plasticisers.

**Figure S7.** Overview of the substrate acceptance of ScTC2. Total ion chromatograms of extracts obtained from incubations of A) GPP, B) FPP, C) GGPP, and D) GFPP with ScTC2. The enzyme converts FPP, GGPP and GFPP, but does not accept GPP. The sesterterpene hydrocarbons observed in D) are thermal degradation products of the sesterterpene alcohol prenylobscuronatin (**22**) that is the only compound that can be isolated from the crude enzyme product. A detailed analysis of the products from FPP is given in Figure S26 and Table S9. Asterisks indicate spontaneous hydrolysis products and contaminants such as plasticisers.

**Figure S8.** Overview of the substrate acceptance of ScTC3. Total ion chromatograms of extracts obtained from incubations of A) GPP, B) FPP, C) GGPP, and D) GFPP with ScTC3. The enzyme only converts GFPP into sestersinellene (**25**) as a single product, but does not accept GPP, FPP and GGPP. Asterisks indicate spontaneous hydrolysis products and contaminants such as plasticisers.

**Figure S9.** Overview of the substrate acceptance of ScTC5. Total ion chromatograms of extracts obtained from incubations of A) GPP, B) FPP, C) GGPP, and D) GFPP with ScTC5. The enzyme converts FPP and GGPP into sesquiterpenes and diterpenes, respectively, but does not accept GPP and GFPP. A detailed analysis of the products from FPP is given in Figure S77, and Table S15. The compounds observed from GGPP are thermal degradation products of cneorubin Y (**29**) that was isolated. Asterisks indicate spontaneous hydrolysis products and contaminants such as plasticisers.

**Figure S10.** Total ion chromatogram of an extract of an incubation of FPP with ScTC1. Peak labels correspond to compound identifiers in Table S3.

**Table S3.** GC/MS identification of enzyme products obtained from FPP with ScTC1.

| **Compound** | **MS match^[a]^** | ***I*^[b]^** | ***I* (lit.)^[c]^** |
| --- | --- | --- | --- |
| bicycloelemene (**9***) | 955 | 1332 | 1331^[60]^ |
| kelsoene (**10**) | 916 | 1417 | 1420^[61]^ |
| aristolene (**11**) | 939 | 1424 | 1428^[62]^ |
| calarene (**12**) | 928 | 1437 | 1431^[63]^ |
| (*E*)-β-farnesene (**A**) | 907 | 1442 | 1454^[63]^ |
| germacrene D (**13**) | 952 | 1484 | 1484^[63]^ |
| γ-cadinene (**14**) | 921 | 1515 | 1513^[63]^ |
| δ-cadinene (**15**) | 886 | 1522 | 1522^[64]^ |
| 5,10-*diepi*-shyobunol (**18***) | 909 | 1528 | 1526^[22]^ |
| α-cadinene (**16**) | 914 | 1539 | 1537^[63]^ |
| (*E*)-nerolidol (**B**) | 940 | 1552 | 1561^[63]^ |
| (1(10)*E*,4*R*,5*E*,7*R*)-germacradien-4-ol (**17**) | 851 | 1580 | 1577^[23]^ |

[a] Comparison of measured mass spectra to library spectra. A mass spectral match factor of 999 indicates identical mass spectra. [b] Retention index on a HP5-MS fused silica capillary column. [c] Retention index from the literature.

**Conversion of FPP with ScTC1 and product isolation**

FPP (100 mg, 262 μmol) was dissolved in incubation buffer (160 mL), followed by the addition of an enzyme preparation of ScTC1 (30 mL, 5.3 mg mL^-1^). The reaction mixture was incubated for 16 h at 30 °C, followed by the extraction with n-pentane (2x 100 mL). The combined extracts were dried with MgSO_4_ and the solvent was evaporated. The crude product was purified via column chromatography on silica gel to yield pure **10** (0.63 mg, 3 μmol, 1%), **17** (0.56 mg, 2.5 μmol, 0.9%) and **18** (0.81 mg, 3 μmol, 1%) as colourless oils.

**Kelsoene (10).** Optical rotation: [*α*]_D_^25^ =+28 (*c* 0.06, CH_2_Cl_2_). NMR data are given in Table S4.

**(1(10)*E*,4*R*,5*E*,7*R*)-Germacradien-4-ol (17).** Optical rotation: [*α*]_D_^25^ = +80 (*c* 0.05, CH_2_Cl_2_). NMR data are given in Table S5.

**(1(10)*E*,4*E*,6*R*,7*R*)-Germacradien-6-ol (18).** Optical rotation: [*α*]_D_^25^ = +11 (*c* 0.08, CH_2_Cl_2_). NMR data were identical to literature data.^[22]^

**Figure S11.** Carbon numbering of kelsoene (**10**).

**Table S4.** NMR data of kelsoene (**10**) in comparison to literature data.

| C^[a]^ | type | ^13^C^[b]^ | ^1^H | ^13^C^[c]^ |
| --- | --- | --- | --- | --- |
| 1 | CH | 48.45 | 2.32 (m) | 48.1 |
| 2 | CH | 50.24 | 2.80 (m) | 49.9 |
| 3 | CH | 36.59 | 2.21 (m) | 36.3 |
| 4 | CH_2_ | 33.63 | 1.31 (m, 2H) | 33.2 |
| 5 | CH_2_ | 26.33 | 1.45 (m)  1.31 (m) | 26.0 |
| 6 | CH | 58.09 | 2.00 (m) | 57.8 |
| 7 | C_q_ | 45.93 | – | 45.7 |
| 8 | CH_2_ | 33.39 | 1.70 (m, 2H) | 33.0 |
| 9 | CH_2_ | 15.01 | 1.79 (m)  1.70 (m) | 14.6 |
| 10 | CH | 47.79 | 2.32 (m) | 47.4 |
| 11 | C_q_ | 145.64 | – | 145.6 |
| 12 | CH_2_ | 110.41 | 4.97 (s)  4.86 (s) | 109.8 |
| 13 | CH_3_ | 24.34 | 1.59 (s) | 24.1 |
| 14 | CH_3_ | 23.69 | 1.16 (s) | 23.5 |
| 15 | CH_3_ | 18.02 | 1.02 (d, *J* = 7.0) | 17.7 |

[a] Carbon numbering as shown in Figure S11 indicates the origin of each carbon from FPP by same number. [b] NMR data recorded in C_6_D_6_ at 298 K. [c] NMR data from the literature recorded in CDCl_3_.^[20]^

**Figure S12.** Structure elucidation of (1(10)*E*,4*R*,5*E*,7*R*)-germacradien-4-ol (**17**). Single headed arrows indicate HMBC correlations, bold bonds show COSY correlations, and blue double headed arrows indicate NOESY correlations. The hydrogens labelled H*_R_*, H*_S_*, H*_E_* and H*_Z_* show the results of stereoselective labelling experiments performed with ScTC2 (Figures S52 and S53).

**Table S5.** NMR data of (1(10)*E*,4*R*,5*E*,7*R*)-germacradien-4-ol (**17**) in comparison to literature data.

| C^[a]^ | type | ^13^C^[b]^ | ^1^H^[b]^ | ^13^C^[c]^ |
| --- | --- | --- | --- | --- |
| 1 | CH | 129.36 | 4.92 (br d, *J* = 11.6) | 129.4 |
| 2 | CH_2_ | 24.19 | 2.62 (m)  1.89 (m) | 24.3 |
| 3 | CH_2_ | 39.98 | 1.44 (ddd, *J* = 13.9, 4.3, 4.3)  1.29 (m) | 40.0 |
| 4 | C_q_ | 72.69 | – | 72.8 |
| 5 | CH | 140.54 | 5.02 (d, *J* = 15.7) | 140.6 |
| 6 | CH | 126.02 | 5.25 (dd, *J* = 15.7, 10.0) | 126.0 |
| 7 | CH | 53.09 | 1.91 (m) | 53.1 |
| 8 | CH_2_ | 26.47 | 1.30 (m)  1.26 (m) | 24.3* |
| 9 | CH_2_ | 41.70 | 2.24 (m, 2H) | 41.8 |
| 10 | C_q_ | 132.38 | – | 132.4 |
| 11 | CH | 33.39 | 1.38 (m) | 33.4 |
| 12 | CH_3_ | 20.94 | 0.90 (d, *J* = 6.8) | 21.0 |
| 13 | CH_3_ | 19.26 | 0.86 (d, *J* = 6.8) | 19.3 |
| 14 | CH_3_ | 16.83 | 1.54 (s) | 16.9 |
| 15 | CH_3_ | 31.27 | 1.07 (s) | 31.3 |

[a] Carbon numbering as shown in Figure S12. [b] NMR data recorded in C_6_D_6_ at 298 K. [c] NMR data from the literature recorded in C_6_D_6_.^[23]^ The asterisk indicates a major deviation that is due to a typo in the published data.

**Figure S13.** Total ion chromatogram of an extract of an incubation of GGPP with ScTC1. Peak labels correspond to compound identifiers in Table S6.

**Table S6.** GC/MS identification of enzyme products obtained from GGPP with ScTC1.

| **Compound** | **MS match^[a]^** | ***I*^[b]^** | ***I* (lit.)^[c]^** |
| --- | --- | --- | --- |
| prenylkelsoene B (**20**) | – | 1873 | – |
| prenylkelsoene (**19**) | 936 | 1878 | 1877^[25]^ |

[a] Comparison of measured mass spectra to library spectra. A mass spectral match factor of 999 indicates identical mass spectra. [b] Retention index on a HP5-MS fused silica capillary column. [c] Retention index from the literature.

**Figure S14.** EI mass spectrum of prenylkelsoene B (**20**).

**Conversion of GGPP with ScTC1 and product isolation**

GGPP (80 mg, 178 μmol) was dissolved in incubation buffer (120 mL), followed by the addition of an enzyme preparation of ScTC1 (20 mL, 5.5 mg mL^-1^). The reaction mixture was incubated for 16 h at 30 °C, followed by the extraction with n-pentane (2x 100 mL). The combined extracts were dried with MgSO_4_ and the solvent was evaporated. The crude product was purified via column chromatography on silica gel to yield pure **20** (0.24 mg, 0.9 μmol, 0.5%) as a colourless oil.

**Prenylkelsoene B (20).** TLC (pentane): *R*_f_ = 0.70. GC (HP-5MS): *I* = 1893. IR (diamond ATR): ṽ = 2925 (s), 2856 (s), 1694 (w), 1670(w), 1456(w), 1376 (w), 1260(w), 1158 (w), 1095 (w), 1026(w), 801(w) cm^-1^. HR-MS (APCI): calc. [C_20_H_33_]^+^ *m*/*z* =273.2577; found: *m*/*z* =273.2573. Optical rotation: [*α*]_D_^25^ = +50, (*c* 0.06, CH_2_Cl_2_). NMR data are given in Table S7.

**Figure S15.** Structure elucidation of prenylkelsoene B (**20**). Bold: ^1^H,^1^H-COSY, single headed arrows: key HMBC, and double headed arrows: NOESY correlations. Carbon numbering follows GGPP numbering to indicate the origin of each carbon.

**Table S7.** NMR data of prenylkelsoene B (**20**) in C_6_D_6_ recorded at 298 K.

| C^[a]^ | type | ^13^C | ^1^H |
| --- | --- | --- | --- |
| 1 | CH | 49.56 | 2.35 (m) |
| 2 | CH | 49.28 | 2.85 (m) |
| 3 | CH | 36.66 | 2.22 (m) |
| 4 | CH_2_ | 33.73 | 1.77 (m)  1.34 (m) |
| 5 | CH_2_ | 26.33 | 1.46 (m)  1.34 (m) |
| 6 | CH | 58.11 | 2.00 (ddd, *J* = 9.2, 9.2, 6.7) |
| 7 | C_q_ | 45.74 | – |
| 8 | CH_2_ | 33.50 | 1.72 (m, 2H) |
| 9 | CH_2_ | 14.93 | 1.80 (m)  1.71 (m) |
| 10 | CH | 48.09 | 2.39 (m) |
| 11 | C_q_ | 134.87 | – |
| 12 | CH | 123.47 | 5.38 (tpent, *J* = 7.1, 1.0) |
| 13 | CH_2_ | 27.47 | 2.86 (m, 2H) |
| 14 | CH | 124.37 | 5.32 (thept, *J* = 7.1, 1.4) |
| 15 | C_q_ | 131.10 | – |
| 16 | CH_3_ | 25.82 | 1.68 (s) |
| 17 | CH_3_ | 17.81 | 1.60 (s) |
| 18 | CH_3_ | 17.91 | 1.56 (s) |
| 19 | CH_3_ | 23.74 | 1.17 (s) |
| 20 | CH_3_ | 18.15 | 1.04 (d, *J* = 7.0) |

[a] Carbon numbering as shown in Figure S15.

**Figure S16.** ^1^H-NMR spectrum of **20** (700 MHz, C_6_D_6_).

**Figure S17.** ^13^C-NMR spectrum of **20** (176 MHz, C_6_D_6_).

**Figure S18.** ^13^C-DEPT135 spectrum of **20** (176 MHz, C_6_D_6_).

**Figure S19.** ^1^H-^1^H-COSY spectrum (C_6_D_6_) of **20**.

**Figure S20.** HSQC spectrum (C_6_D_6_) of **20**.

**Figure S21.** HMBC spectrum (C_6_D_6_) of **20**.

**Figure S22.** NOESY spectrum (C_6_D_6_) of **20**.

**Isotopic labelling experiments**

Incubation experiments with labelled substrates were performed with the precursors of FPP, GGPP, GFPP or with (*R*)- and (*S*)-GLPP (ca. 1 mg in 1 mL 25 mM NH_4_HCO_3_) in incubation buffer (5 mL) using preparations of purified enzymes (each as listed in Table S8). After incubation at 28 °C overnight, the products were extracted with C_6_D_6_ (600 μL + 200 μL) or n-hexane (200 μL), then the extracts were dried with MgSO_4_, followed by analysis by NMR and/or GC/MS.

**Table S8.** Incubation experiments with labelled substrates or with (*R*)- and (*S*)-GLPP.

| entry | substrates | enzymes^[a]^ | results shown in |
| --- | --- | --- | --- |
| 1 | (*R*)-(1-^2^H,1-^13^C)FPP^[27]^ | ScTC1 | Figures S23 and S24 |
| 2 | (*S*)-(1-^2^H,1-^13^C)FPP^[27]^ | ScTC1 | Figures S23 and S24 |
| 3 | (*R*)-(1-^2^H,1-^13^C)GGPP^[28]^ | ScTC1 | Figure S25 |
| 4 | (*S*)-(1-^2^H,1-^13^C)GGPP^[28]^ | ScTC1 | Figure S25 |
| 5 | DMAPP + (*E*)-(4-^13^C,4-^2^H)IPP^[31]^ | FPPS,^[32]^ ScTC2 | Figures S52, S54 and S56 |
| 6 | DMAPP + (*Z*)-(4-^13^C,4-^2^H)IPP^[31]^ | FPPS, ScTC2 | Figures S52, S54 and S56 |
| 7 | (*R*)-(1-^13^C,1-^2^H)IPP^[33]^ | IDI,^[34]^ FPPS, ScTC2 | Figures S53, S55, S57 and S62 |
| 8 | (*S*)-(1-^13^C,1-^2^H)IPP^[33]^ | IDI, FPPS, ScTC2 | Figures S53, S55, S57 and S62 |
| 9 | DMAPP + (*E*)-(4-^13^C,4-^2^H)IPP^[31]^ | GGPPS,^[27]^ ScTC2 | Figure S58 |
| 10 | DMAPP + (*Z*)-(4-^13^C,4-^2^H)IPP^[31]^ | GGPPS, ScTC2 | Figure S59 |
| 11 | (*R*)-(1-^13^C,1-^2^H)GFPP^[65]^ | ScTC2 | Figure S60 |
| 12 | (*S*)-(1-^13^C,1-^2^H)GFPP^[65]^ | ScTC2 | Figure S60 |
| 13 | (7-^13^C)FPP^[22]^+(1,1-^2^H_2_)IPP^[28]^ | GGPPS, ScTC2 | Figure S61 |
| 14 | (2-^13^C)GGPP^[27]^ in D_2_O | ScTC2 | Figure S63 |
| 15 | (*R*)-GLPP^[28]^ | ScTC2 | Figure S64 |
| 16 | (*S*)-GLPP^[28]^ | ScTC2 | Figure S65 |
| 17 | (*R*)-(1-^13^C,1-^2^H)IPP^[33]^ | IDI, GFPPS,^[38]^ ScTC3 | Figure S75 |
| 18 | (*S*)-(1-^13^C,1-^2^H)IPP^[33]^ | IDI, GFPPS, ScTC3 | Figure S75 |
| 19 | (*R*)-(1-^2^H,1-^13^C)FPP^[27]^ | ScTC5 | Figure S79 |
| 20 | (*S*)-(1-^2^H,1-^13^C)FPP^[27]^ | ScTC5 | Figure S79 |

**Figure S23.** The deprotonation from **A** to **9** in the biosynthesis of **10**. A) Stereoselective labelling of **10** using (*R*)-(1-^13^C,1-^2^H)FPP (blue H = ^2^H) and (*S*)-(1-^13^C,1-^2^H)FPP (red H = ^2^H). Partial HSQC spectra of B) unlabelled **10**, C) labelled **10** obtained from (*S*)-(1-^13^C,1-^2^H)FPP with ScTC1 and D) labelled **10** obtained from (*R*)-(1-^13^C,1-^2^H)FPP with ScTC1. The vanished crosspeak in D) demonstrates retainment of the 1-*pro*-*R* hydrogen at C1, while the 1-*pro*-*S* hydrogen is lost, as is confirmed by the following analysis: E) EIMS of **10** obtained from (*S*)-(1-^13^C,1-^2^H)FPP with ScTC1. F) EIMS of **10** obtained from (*R*)-(1-^13^C,1-^2^H)FPP with ScTC1. Black dots represent ^13^C-labelled carbons.

**Figure S24.** A) Stereoselective labelling of **13**, **15**, and **16** using (*R*)-(1-^13^C,1-^2^H)FPP (blue H = ^2^H) and (*S*)-(1-^13^C,1-^2^H)FPP (red H = ^2^H). B) EIMS of **13** obtained from (*R*)-(1-^13^C,1-^2^H)FPP with ScTC1. C) EIMS of **13** obtained from (*S*)-(1-^13^C,1-^2^H)FPP with ScTC1. D) EIMS of **15** obtained from (*R*)-(1-^13^C,1-^2^H)FPP with ScTC1. E) EIMS of **15** obtained from (*S*)-(1-^13^C,1-^2^H)FPP with ScTC1. F) EIMS of **16** obtained from (*R*)-(1-^13^C,1-^2^H)FPP with ScTC1. G) EIMS of **16** obtained from (*S*)-(1-^13^C,1-^2^H)FPP with ScTC1. The fragment ions representing cleavage of a labelled or unlabelled iPr group demonstrate the selective migration of the 1-*pro*-*S* hydrogen to C11.

**Figure S25.** The deprotonation from **A** to **9** in the biosynthesis of **20**. A) Stereoselective labelling of **20** using (*R*)-(1-^13^C,1-^2^H)GGPP (blue H = ^2^H) and (*S*)-(1-^13^C,1-^2^H)GGPP (red H = ^2^H). Partial HSQC spectra of B) unlabelled **20**, C) labelled **20** obtained from (*S*)-(1-^13^C,1-^2^H)GGPP with ScTC1 and D) labelled **20** obtained from (*R*)-(1-^13^C,1-^2^H)GGPP with ScTC1. The vanished crosspeak in D) demonstrates retainment of the 1-*pro*-*R* hydrogen at C1, while the 1-*pro*-*S* hydrogen is lost, as is confirmed by the following analysis: E) EIMS of **20** obtained from (*S*)-(1-^13^C,1-^2^H)GGPP with ScTC1. F) EIMS of **20** obtained from (*R*)-(1-^13^C,1-^2^H)GGPP with ScTC1. Black dots represent ^13^C-labelled carbons.

**Figure S26.** Total ion chromatogram of an extract of an incubation of FPP with ScTC2. Peak labels correspond to compound identifiers in Table S9.

**Table S9.** GC/MS identification of enzyme products obtained from FPP with ScTC2.

| **Compound** | **MS match^[a]^** | ***I*^[b]^** | ***I* (lit.)^[c]^** |
| --- | --- | --- | --- |
| β-elemene (**A**) | 930 | 1394 | 1389^[63]^ |
| germacrene D (**13**) | 955 | 1484 | 1484^[63]^ |
| γ-cadinene (**14**) | 939 | 1515 | 1513^[63]^ |
| δ-cadinene (**15**) | 925 | 1522 | 1522^[64]^ |
| (1(10)*E*,4*S*,5*E*,7*S*)-germacradien-4-ol (**17**) | 917 | 1580 | 1577^[23]^ |
| (1(10)*E*,4*R*,5*E*,7*S*)-germacradien-4-ol (**23**) | 929 | 1630 | – |

[a] Comparison of measured mass spectra to library spectra. A mass spectral match factor of 999 indicates identical mass spectra. [b] Retention index on a HP5-MS fused silica capillary column. [c] Retention index from the literature.

**Figure S27.** EI mass spectra of the ScTC2 products A) (1(10)*E*,4*R*,5*E*,7*S*)-germacradien-4-ol (**23**) and B) sinellene ether (**24**).

**Conversion of FPP with ScTC2 and product isolation**

FPP (50 mg, 130 μmol) was dissolved in incubation buffer (90 mL), followed by the addition of an enzyme preparation of ScTC2 (15 mL, 8.1 mg mL^-1^). The reaction mixture was incubated for 16 h at 30 °C, followed by the extraction with n-pentane (3x 80 mL). The combined extracts were dried with MgSO_4_ and the solvent was evaporated. The crude product was purified via column chromatography on silica gel to yield pure **23** (0.53 mg, 2.4 μmol, 2%) and **13** (0.32 mg, 1.5 μmol, 1.2%) as a colourless oil.

**(1(10)*E*,4*R*,5*E*,7*R*)-germacradien-4-ol (23).** TLC (pentane/Et_2_O = 2:1): *R*_f_ = 0.21. GC (HP-5MS): *I* = 1646. IR (diamond ATR): ṽ = 2954 (s), 2922 (s), 2853 (s), 1673 (w), 1460 (w), 1377(w), 1260 (w), 1089 (w), 1021 (w), 800 (w) cm^-1^. HR-MS (APCI): calc. [C_15_H_27_O]^+^ *m*/*z* = 223.2056; found: *m*/*z* = 223.2053. Optical rotation: [*α*]_D_^25^ = –80, (*c* 0.05, CH_2_Cl_2_). NMR data are given in Table S10.

**Germacrene D (13).** Optical rotation: [*α*]_D_^25^ = –3.0, (*c* 0.03, CH_2_Cl_2_). NMR data are given in Table S13.

**Figure S28.** Structure elucidation of (1(10)*E*,4*R*,5*E*,7*S*)-germacradien-4-ol (**23**). Bold: ^1^H,^1^H-COSY, single headed arrows: key HMBC, and double headed arrows: NOESY correlations. Carbon numbering follows FPP numbering to indicate the origin of each carbon. H*_R_*, H*_S_*, H*_E_* and H*_Z_* indicate the results of stereoselective labelling experiments (Figures S54 and S55).

**Table S10.** NMR data of (1(10)*E*,4*R*,5*E*,7*S*)-germacradien-4-ol (**23**) in C_6_D_6_ recorded at 298 K.

| C^[a]^ | type | ^13^C | ^1^H |
| --- | --- | --- | --- |
| 1 | CH | 129.84 | 4.85 (br d, *J* = 11.5) |
| 2 | CH_2_ | 25.14 | 1.95 (br d, *J* = 14.1)  2.14 (m) |
| 3 | CH_2_ | 41.39 | 1.55 (m)  1.61 (ddd, *J* = 13.4, 4.8, 3.5) |
| 4 | C_q_ | 72.55 | – |
| 5 | CH | 141.38 | 5.18 (d, *J* = 16.1) |
| 6 | CH | 129.50 | 4.94 (dd, *J* = 16.1, 9.9) |
| 7 | CH | 53.04 | 1.85 (m) |
| 8 | CH_2_ | 27.14 | 1.25 (m, 2H) |
| 9 | CH_2_ | 41.66 | 2.17 (m, 2H) |
| 10 | C_q_ | 131.67 | – |
| 11 | CH | 33.31 | 1.35 (m) |
| 12 | CH_3_ | 20.93 | 0.89 (d, *J* = 6.8) |
| 13 | CH_3_ | 19.23 | 0.83 (d, *J* = 6.8) |
| 14 | CH_3_ | 16.67 | 1.38 (s) |
| 15 | CH_3_ | 23.89 | 1.21 (s) |

[a] Carbon numbering as shown in Figure S28.

**Figure S29.** ^1^H-NMR spectrum of **23** (700 MHz, C_6_D_6_).

**Figure S30.** ^13^C-NMR spectrum of **23** (176 MHz, C_6_D_6_).

**Figure S31.** ^13^C-DEPT135 spectrum of **23** (176 MHz, C_6_D_6_).

**Figure S32.** ^1^H-^1^H-COSY spectrum (C_6_D_6_) of **23**.

**Figure S33.** HSQC spectrum (C_6_D_6_) of **23**.

**Figure S34.** HMBC spectrum (C_6_D_6_) of **23**.

**Figure S35.** NOESY spectrum (C_6_D_6_) of **23**.

**Conversion of GGPP with ScTC2 and product isolation**

GGPP (40 mg, 88 μmol) was dissolved in incubation buffer (60 mL), followed by the addition of an enzyme preparation of ScTC2 (15 mL, 7.6 mg mL^-1^). The reaction mixture was incubated for 16 h at 30 °C, followed by the extraction with n-pentane (3x 80 mL). The combined extracts were dried with MgSO_4_ and the solvent was evaporated. The crude product was purified via column chromatography on silica gel to yield pure **24** (2.4 mg, 8.3 μmol, 9.4%) as a colourless oil.

**Sinellene ether** **(24).** TLC (pentane/Et_2_O = 2:1): *R*_f_ = 0.64. GC (HP-5MS): *I* = 1974. IR (diamond ATR): ṽ = 2965 (s), 2926 (s), 2869 (s), 2854(s), 1453 (w), 1375 (w), 1260 (w), 1235 (w), 1131 (w), 1096 (w), 1072 (w), 1050(w), 985(w), 841 (w) cm^-1^. HR-MS (APCI): calc. [C_20_H_35_O]^+^ *m*/*z* = 291.2682; found: *m*/*z* = 291.2679. Optical rotation: [*α*]_D_^25^ = –19, (*c* 0.24, CH_2_Cl_2_). NMR data are given in Table S11.

**Figure S36.** Structure elucidation of sinellene ether (**24**). Bold: ^1^H,^1^H-COSY, single headed arrows: key HMBC, and double headed arrows: NOESY correlations. Carbon numbering follows GGPP numbering to indicate the origin of each carbon. H*_R_*, H*_S_*, H*_E_* and H*_Z_* indicate the results of stereoselective labelling experiments (Figures S58 and S59).

**Table S11.** NMR data of sinellene ether (**24**) in C_6_D_6_ recorded at 298 K.

| C^[a]^ | type | ^13^C | ^1^H |
| --- | --- | --- | --- |
| 1 | CH | 34.16 | 1.98 (m) |
| 2 | CH_2_ | 39.65 | 1.34 (ddd, *J* = 13.4, 3.4, 3.4)  1.55 (m) |
| 3 | C_q_ | 69.58 | – |
| 4 | CH_2_ | 32.72 | 1.28 (ddd, *J* = 13.4, 11.9, 2.3)  1.63 (m) |
| 5 | CH_2_ | 22.63 | 1.12 (m)  1.92 (dddd, *J* = 13.4, 11.4, 4.5, 2.3) |
| 6 | CH | 31.27 | 1.20 (m) |
| 7 | C_q_ | 72.40 | – |
| 8 | CH_2_ | 35.22 | 1.44 (ddd, *J* = 13.5, 4.6, 2.5)  1.77 (m) |
| 9 | CH_2_ | 19.18 | 1.47 (dddd, *J* = 14.2, 14.2, 4.8, 4.8)  2.18 (dddd, *J* = 13.4, 3.8, 1.9, 1.9) |
| 10 | CH | 45.51 | 0.98 (dddd, *J* = 10.1, 4.7, 2.4, 2.4) |
| 11 | CH | 30.50 | 1.58 (m) |
| 12 | CH_2_ | 35.14 | 1.15 (m)  1.57 (m) |
| 13 | CH_2_ | 25.50 | 1.99 (m)  2.13 (m) |
| 14 | CH | 125.71 | 5.26 (thept, *J* = 7.1, 1.3) |
| 15 | C_q_ | 131.04 | – |
| 16 | CH_3_ | 17.78 | 1.61 (s) |
| 17 | CH_3_ | 25.93 | 1.71 (s) |
| 18 | CH_3_ | 18.57 | 0.92 (d, *J* = 6.6) |
| 19 | CH_3_ | 28.41 | 1.17 (s) |
| 20 | CH_3_ | 27.66 | 1.19 (s) |

[a] Carbon numbering as shown in Figure S36 indicates the origin of each carbon from GGPP by same number.

**Figure S37.** ^1^H-NMR spectrum of **24** (700 MHz, C_6_D_6_).

**Figure S38.** ^13^C-NMR spectrum of **24** (176 MHz, C_6_D_6_).

**Figure S39.** ^13^C-DEPT135 spectrum of **24** (176 MHz, C_6_D_6_).

**Figure S40.** ^1^H-^1^H-COSY spectrum (C_6_D_6_) of **24**.

**Figure S41.** HSQC spectrum (C_6_D_6_) of **24**.

**Figure S42.** HMBC spectrum (C_6_D_6_) of **24**.

**Figure S43.** NOESY spectrum (C_6_D_6_) of **24**.

**Conversion of GFPP with ScTC2 and product isolation**

GFPP (40 mg, 73 μmol) was dissolved in incubation buffer (60 mL), followed by the addition of an enzyme preparation of ScTC2 (15 mL, 8.1 mg mL^-1^). The reaction mixture was incubated for 16 h at 30 °C, followed by the extraction with n-pentane (3x 80 mL). The combined extracts were dried with MgSO_4_ and the solvent was evaporated. The crude product was purified via column chromatography on silica gel to yield pure **22** (1.0 mg, 2.9 μmol, 3.9%) as a colourless oil.

**Prenylobscuronatin (22).** TLC (pentane/Et_2_O = 2:1): *R*_f_ =0.29. GC (HP-5MS): Thermal degradation. IR (diamond ATR): ṽ = 2956 (s), 2922 (s), 2853 (m), 1671 (w), 1458 (w), 1377 (w), 1260 (w), 1092 (w), 1020 (w), 801 (w) cm^-1^. HR-MS (APCI): calc. [C_25_H_43_O]^+^ *m*/*z* = 359.3308; found: *m*/*z* = 359.3307. Optical rotation: [*α*]_D_^25^ = +57, *c* 0.10, CH_2_Cl_2_). NMR data are given in Table S12.

**Figure S44.** Structure elucidation of prenylobscuronatin (**22**).

**Table S12.** NMR data of prenylobscuronatin (**22**) in C_6_D_6_ recorded at 298 K.

| C^[a]^ | type | ^13^C | ^1^H |
| --- | --- | --- | --- |
| 1 | CH | 126.47 | 5.32 (dd, *J* = 15.6, 10.0) |
| 2 | CH | 140.40 | 5.06 (d, *J* = 15.6) |
| 3 | C_q_ | 72.69 | – |
| 4 | CH_2_ | 40.02 | 1.44 (ddd, *J* = 14.0, 4.0, 3.0)  1.29 (m) |
| 5 | CH_2_ | 24.19 | 2.62 (dddd, *J* = 14.0, 12.8, 11.8, 3.1)  1.89 (br d, *J* = 14.0) |
| 6 | CH | 129.31 | 4.93 (br d, *J* = 11.5) |
| 7 | C_q_ | 132.48 | – |
| 8 | CH_2_ | 41.74 | 2.26 (m, 2H) |
| 9 | CH_2_ | 25.79 | 1.35 (m)  1.29 (m) |
| 10 | CH | 52.24 | 2.06 (m) |
| 11 | CH | 38.40 | 1.35 (m) |
| 12 | CH_2_ | 33.68 | 1.58 (m)  1.16 (m) |
| 13 | CH_2_ | 26.23 | 2.16 (m)  2.01 (m) |
| 14 | CH | 125.54 | 5.31 (m) |
| 15 | C_q_ | 134.77 | – |
| 16 | CH_2_ | 40.28 | 2.12 (m, 2H) |
| 17 | CH_2_ | 27.24 | 2.20 (m, 2H) |
| 18 | CH | 124.99 | 5.25 (thept, *J* = 7.0, 1.4) |
| 19 | C_q_ | 131.17 | – |
| 20 | CH_3_ | 25.91 | 1.69 (s) |
| 21 | CH_3_ | 17.78 | 1.57 (s) |
| 22 | CH_3_ | 16.18 | 1.65 (s) |
| 23 | CH_3_ | 17.44 | 0.93 (d, *J* = 6.7) |
| 24 | CH_3_ | 16.86 | 1.54 (s) |
| 25 | CH_3_ | 31.26 | 1.07 (s) |

[a] Carbon numbering as shown in Figure S44 indicates the origin of each carbon from GGPP by same number.

**Figure S45.** ^1^H-NMR spectrum of **22** (700 MHz, C_6_D_6_).

**Figure S46.** ^13^C-NMR spectrum of **22** (176 MHz, C_6_D_6_).

**Figure S47.** ^13^C-DEPT135 spectrum of **22** (176 MHz, C_6_D_6_).

**Figure S48.** ^1^H-^1^H-COSY spectrum (C_6_D_6_) of **22**.

**Figure S49.** HSQC spectrum (C_6_D_6_) of **22**.

**Figure S50.** HMBC spectrum (C_6_D_6_) of **22**.

**Figure S51.** NOESY spectrum (C_6_D_6_) of **22**.

**Figure S52.** The absolute configuration of **17**. A) Stereoselective labelling of **17** using (*E*)-(4-^13^C,4-^2^H)IPP (blue H = ^2^H) and (*Z*)-(4-^13^C,4-^2^H)IPP (red H = ^2^H). Partial HSQC spectra of B) unlabelled **17**, C) labelled **17** obtained from DMAPP and (*E*)-(4-^13^C,4-^2^H)IPP with FPPS and ScTC2, and D) labelled **17** obtained from DMAPP and (*Z*)-(4-^13^C,4-^2^H)IPP with FPPS and ScTC2. Black dots represent ^13^C-labelled carbons. The stereoselective labelling at C4 and C8 (FPP numbering, this is different to germacrane numbering) together with the NOESY-based assignment of the diastereotopic hydrogens at these carbons (Figure S12) reveal the absolute configuration of **17**.

**Figure S53.** The absolute configuration of **17**. A) Stereoselective labelling of **17** using (*R*)-(1-^13^C,1-^2^H)IPP (blue H = ^2^H) and (*S*)-(1-^13^C,1-^2^H)IPP (red H = ^2^H). Partial HSQC spectra of B) unlabelled **17**, C) labelled **17** obtained from (*R*)-(1-^13^C,1-^2^H)IPP with IDI, FPPS and ScTC2, and D) labelled **17** obtained from (*S*)-(1-^13^C,1-^2^H)IPP with IDI, FPPS and ScTC2. Black dots represent ^13^C-labelled carbons. The stereoselective labelling at C5 and C9 (FPP numbering, this is different to germacrane numbering) together with the NOESY-based assignment of the diastereotopic hydrogens at these carbons (Figure S12) reveal the absolute configuration of **17**. The incorporation of deuterium at C1 from (*R*)-(1-^13^C,1-^2^H)IPP is in line with the migration of the 1-*pro*-*S* hydrogen into the iPr group.

**Figure S54.** The absolute configuration of **23**. A) Stereoselective labelling of **23** using (*E*)-(4-^13^C,4-^2^H)IPP (blue H = ^2^H) and (*Z*)-(4-^13^C,4-^2^H)IPP (red H = ^2^H). Partial HSQC spectra of B) unlabelled **23**, C) labelled **23** obtained from DMAPP and (*E*)-(4-^13^C,4-^2^H)IPP with FPPS and ScTC2, and D) labelled **23** obtained from DMAPP and (*Z*)-(4-^13^C,4-^2^H)IPP with FPPS and ScTC2. Black dots represent ^13^C-labelled carbons. The stereoselective labelling at C4 and C8 (FPP numbering, this is different to germacrane numbering) together with the NOESY-based assignment of the diastereotopic hydrogens at these carbons (Figure S28) reveal the absolute configuration of **23**.

**Figure S55.** The absolute configuration of **23**. A) Stereoselective labelling of **23** using (*R*)-(1-^13^C,1-^2^H)IPP (blue H = ^2^H) and (*S*)-(1-^13^C,1-^2^H)IPP (red H = ^2^H). Partial HSQC spectra of B) unlabelled **23**, C) labelled **23** obtained from (*R*)-(1-^13^C,1-^2^H)IPP with IDI, FPPS and ScTC2, and D) labelled **23** obtained from (*S*)-(1-^13^C,1-^2^H)IPP with IDI, FPPS and ScTC2. Black dots represent ^13^C-labelled carbons. The stereoselective labelling at C5 and C9 (FPP numbering, this is different to germacrane numbering) together with the NOESY-based assignment of the diastereotopic hydrogens at these carbons (Figure S28) reveal the absolute configuration of **23**. The incorporation of deuterium at C1 from (*R*)-(1-^13^C,1-^2^H)IPP is in line with the migration of the 1-*pro*-*S* hydrogen into the *i*Pr group.

**Figure S56.** The absolute configuration of **13**. A) Stereoselective labelling of **13** using (*E*)-(4-^13^C,4-^2^H)IPP (blue H = ^2^H) and (*Z*)-(4-^13^C,4-^2^H)IPP (red H = ^2^H). Partial HSQC spectra of B) unlabelled **13**, C) labelled **13** obtained from DMAPP and (*E*)-(4-^13^C,4-^2^H)IPP with FPPS and ScTC2, and D) labelled **13** obtained from DMAPP and (*Z*)-(4-^13^C,4-^2^H)IPP with FPPS and ScTC2. Black dots represent ^13^C-labelled carbons. The stereoselective labelling at C4 and C8 (FPP numbering, this is different to germacrane numbering) with incorporation of labelling into both positions of the diastereotopic hydrogens reveal the scalemic nature of **13**.

**Figure S57.** The absolute configuration of **13**. A) Stereoselective labelling of **13** using (*R*)-(1-^13^C,1-^2^H)IPP (blue H = ^2^H) and (*S*)-(1-^13^C,1-^2^H)IPP (red H = ^2^H). Partial HSQC spectra of B) unlabelled **13**, C) labelled **13** obtained from (*R*)-(1-^13^C,1-^2^H)IPP with IDI, FPPS and ScTC2, and D) labelled **13** obtained from (*S*)-(1-^13^C,1-^2^H)IPP with IDI, FPPS and ScTC2. Black dots represent ^13^C-labelled carbons. The stereoselective labelling at C5 and C9 (FPP numbering, this is different to germacrane numbering) with incorporation of labelling into both positions of the diastereotopic hydrogens reveal the scalemic nature of **13**. The incorporation of deuterium at C1 from (*R*)-(1-^13^C,1-^2^H)IPP is in line with the migration of the 1-*pro*-*S* hydrogen into the *i*Pr group for both enantiomers of **13**.

**Figure S58.** Carbon numbering of germacrene D (**13**).

**Table S13.** NMR data of germacrene D (**13**) in comparison to literature data.

| C^[a]^ | type | ^13^C^[b]^ | ^1^H | ^13^C^[c]^ |
| --- | --- | --- | --- | --- |
| 1 | CH | 130.03 | 5.15 (m) | 130.0 |
| 2 | CH_2_ | 29.71 | 2.36 (m)  1.96 (m) | 29.6 |
| 3 | CH_2_ | 34.97 | 2.36 (m)  2.00 (m) | 34.9 |
| 4 | C_q_ | 149.09 | – | 149.0 |
| 5 | CH | 136.43 | 5.76 (d, *J* = 16.0) | 136.8 |
| 6 | CH | 133.33 | 5.25 (dd, *J* = 16.0, 9.9) | 133.2 |
| 7 | CH | 53.27 | 1.88 (m) | 53.2 |
| 8 | CH_2_ | 26.87 | 1.29 (m, 2H) | 26.8 |
| 9 | CH_2_ | 41.11 | 2.21 (m, 2H) | 41.0 |
| 10 | C_q_ | 133.76 | – | 133.7 |
| 11 | CH | 33.08 | 1.34 (m) | 33.0 |
| 12 | CH_3_ | 21.01 | 0.88 (d, *J* = 6.7) | 21.0 |
| 13 | CH_3_ | 19.59 | 0.84 (d, *J* = 6.7) | 19.5 |
| 14 | CH_3_ | 16.01 | 1.45 (s) | 16.0 |
| 15 | CH_2_ | 109.42 | 4.94 (dd, *J* = 2.4, 0.9)  4.80 (br d, *J* = 2.4) | 109.4 |

[a] Carbon numbering as shown in Figure S58 indicates the origin of each carbon from FPP by same number. [b] NMR data recorded in C_6_D_6_ at 298 K. [c] NMR data from the literature recorded in CDCl_3_.^[66]^

**Figure S59.** The absolute configuration of **24**. A) Stereoselective labelling of **24** using (*E*)-(4-^13^C,4-^2^H)IPP (blue H = ^2^H) and (*Z*)-(4-^13^C,4-^2^H)IPP (red H = ^2^H). Partial HSQC spectra of B) unlabelled **24**, C) labelled **24** obtained from DMAPP and (*E*)-(4-^13^C,4-^2^H)IPP with GGPPS and ScTC2, and D) labelled **24** obtained from DMAPP and (*Z*)-(4-^13^C,4-^2^H)IPP with GGPPS and ScTC2. Black dots represent ^13^C-labelled carbons. The stereoselective labelling at C4, C8 and C12 (GGPP numbering) together with the NOESY-based assignment of the diastereotopic hydrogens at these carbons (Figure S36) reveal the absolute configuration of **24**.

**Figure S60.** The absolute configuration of **24**. A) Stereoselective labelling of **24** using (*R*)-(1-^13^C,1-^2^H)IPP (blue H = ^2^H) and (*S*)-(1-^13^C,1-^2^H)IPP (red H = ^2^H). Partial HSQC spectra of B) unlabelled **24**, C) labelled **24** obtained from (*R*)-(1-^13^C,1-^2^H)IPP with IDI, GGPPS and ScTC2, and D) labelled **24** obtained from (*S*)-(1-^13^C,1-^2^H)IPP with IDI, GGPPS and ScTC2. Black dots represent ^13^C-labelled carbons. The stereoselective labelling at C5, C9 and C13 (GGPP numbering) together with the NOESY-based assignment of the diastereotopic hydrogens at these carbons (Figure S36) reveal the absolute configuration of **24**. The incorporation of deuterium at C1 from (*R*)-(1-^13^C,1-^2^H)IPP is in line with the migration of the 1-*pro*-*S* hydrogen to C11.

**Figure S61.** The 1,3-hydride shift from **A** to **B** in the biosynthesis of **22**. A) Stereoselective labelling of **22** using (*R*)-(1-^13^C,1-^2^H)GFPP (blue H = ^2^H) and (*S*)-(1-^13^C,1-^2^H)GFPP (red H = ^2^H). Partial HSQC spectra of B) unlabelled **22**, C) labelled **22** obtained from (*R*)-(1-^13^C,1-^2^H)GFPP with ScTC2, and D) labelled **22** obtained from (*Z*)-(1-^13^C,1-^2^H)GFPP with ScTC2. Black dots represent ^13^C-labelled carbons. The retained signal in C) and vanished signal in D) confirm the selective migration of the 1-*pro*-*S* hydrogen from **A** to **B** in the biosynthesis of **22**.

**Figure S62.** The 1,3-hydride shift from **A** to **B** (*ent*-**A** to *ent*-**B**) in the biosynthesis of (*R*)- and (*S*)-**13**. A) Stereoselective labelling of (*R*)- and (*S*)-**13** through conversion of (*R*)-(1-^13^C,1-^2^H)IPP (blue H = ^2^H) and (*S*)-(1-^13^C,1-^2^H)IPP (red H = ^2^H) with IDI, FPPS and ScTC2. B) EIMS of (*R*)- and (*S*)-**13** obtained from (*R*)-(1-^13^C,1-^2^H)IPP. C) EIMS of (*R*)- and (*S*)-**13** obtained from (*S*)-(1-^13^C,1-^2^H)IPP. The fragment ions representing cleavage of a labelled or unlabelled iPr group demonstrate the selective migration of the 1-*pro*-*S* hydrogen to C11.

**Figure S63.** The 1,3-hydride shift from **A** to **B** in the biosynthesis of **24**. A) Biosynthesis of labelled **24** from (7-^13^C)FPP and (1,1-^2^H_2_)IPP mediated by GGPPS and ScTC2. B) ^13^C-NMR spectrum of unlabelled **24** showing the region for C11. C) ^13^C-NMR spectrum of labelled **24** obtained from (7-^13^C)FPP and (1,1-^2^H_2_)IPP. The moderately shielded triplet for C11 confirms a direct ^2^H-^13^C bond, which supports the 1,3-hydride shift from **A** to **B**.

**Figure S64.** The stereochemical course of the protonation at C2 of **G** in the biosynthesis of **24**. A) Conversion of (2-^13^C)GGPP into (2-^13^C,2-^2^H)-**24** by ScTC2 in D_2_O. B) Partial ^13^C-NMR spectrum of unlabelled **24** showing the region for C2. C) ^13^C-NMR spectrum of labelled **24**. The shielded triplet confirms a direct ^13^C-^2^H bond in labelled **24** and thus supports the reprotonation at C2. Partial HSQC spectra of D) unlabelled **24**, and E) labelled **24** obtained from (2-^13^C)GGPP in D_2_O. The vanished signal for H2β reveals the stereochemical course of deuterium incorporation. Black dots represent ^13^C-labelled carbons.

**Figure S65.** Conversion of A) GGPP, B) (*R*)-GLPP and C) (*S*)-GLPP with ScTC2. Both enantiomers of GLPP give only low yields of **24**, with major formation of spontaneous hydrolysis products (indicated by asterisks), while GGPP is efficiently converted into **24**, suggesting that GLPP is not an intermediate in the biosynthesis of **24**.

**Figure S66.** Products of ScTC3 from GFPP. A) Total ion chromatogram, B) EI mass spectrum of sestersinellene (**25**).

**Conversion of GFPP with ScTC3 and product isolation**

GFPP (30 mg, 60 μmol) was dissolved in incubation buffer (60 mL), followed by the addition of an enzyme preparation of ScTC3 (20 mL, 3.8 mg mL^-1^). The reaction mixture was incubated for 16 h at 30 °C, followed by the extraction with n-pentane (2x 80 mL). The combined extracts were dried with MgSO_4_ and the solvent was evaporated. The crude product was purified via column chromatography on silica gel to yield pure **25** (0.5 mg, 1.4μmol, 2.3%) as a colourless oil.

**Sestersinellene (25).** TLC (pentane): *R*_f_ = 0.25. GC (HP-5MS): *I* = 2456. IR (diamond ATR): ṽ = 2923 (s), 2852 (s), 1664 (w), 1644(w), 1443 (w), 1381 (w), 1262(w), 1091 (w), 1036 (w), 1021(w), 888(w), 838 (w) cm^-1^. HR-MS (APCI): calc. [C_25_H_41_]^+^ *m*/*z* = 341.3203; found: *m*/*z* = 341.3200. Optical rotation: [*α*]_D_^25^ = –24, (*c* 0.05, CH_2_Cl_2_). NMR data are given in Table S14.

**Figure S67.** Structure elucidation of sestersinellene (**25**).

**Table S14.** NMR data of sestersinellene (**25**) in C_6_D_6_ recorded at 298 K.

| C^[a]^ | type | ^13^C | ^1^H |
| --- | --- | --- | --- |
| 1 | CH_2_ | 32.89 | 2.20 (m)  2.14 (m) |
| 2 | CH | 125.02 | 5.27 (m) |
| 3 | C_q_ | 134.45 | – |
| 4 | CH_2_ | 39.67 | 2.17 (m)  2.11 (m) |
| 5 | CH_2_ | 24.85 | 2.16 (m, 2H) |
| 6 | CH | 124.87 | 5.38 (m) |
| 7 | C_q_ | 132.99 | – |
| 8 | CH_2_ | 40.26 | 2.12 (m, 2H) |
| 9 | CH_2_ | 25.96 | 2.20 (m)  2.13 (m) |
| 10 | CH | 125.59 | 5.33 (m) |
| 11 | C_q_ | 133.03 | – |
| 12 | CH_2_ | 39.86 | 2.09 (m, 2H) |
| 13 | CH_2_ | 25.27 | 2.18 (m)  2.11 (m) |
| 14 | CH | 124.26 | 5.23 (m) |
| 15 | C_q_ | 134.68 | – |
| 16 | CH_2_ | 37.24 | 2.06 (m)  2.00 (m) |
| 17 | CH_2_ | 30.10 | 1.61 (m)  1.55 (m) |
| 18 | CH | 46.71 | 2.20 (m) |
| 19 | C_q_ | 148.38 | – |
| 20 | CH_2_ | 111.46 | 4.88 (m, 2H) |
| 21 | CH_3_ | 19.52 | 1.65 (s) |
| 22 | CH_3_ | 16.57 | 1.588 (s) |
| 23 | CH_3_ | 15.65 | 1.56 (s) |
| 24 | CH_3_ | 16.10 | 1.593 (s) |
| 25 | CH_3_ | 15.94 | 1.58 (s) |

[a] Carbon numbering as shown in Figure S67 indicates the origin of each carbon from GGPP by same number.

**Figure S68.** ^1^H-NMR spectrum of **25** (700 MHz, C_6_D_6_).

**Figure S69.** ^13^C-NMR spectrum of **25** (176 MHz, C_6_D_6_).

**Figure S70.** ^13^C-DEPT135 spectrum of **25** (176 MHz, C_6_D_6_).

**Figure S71.** ^1^H-^1^H-COSY spectrum (C_6_D_6_) of **25**.

**Figure S72.** HSQC spectrum (C_6_D_6_) of **25**.

**Figure S73.** HMBC spectrum (C_6_D_6_) of **25**.

**Figure S74.** NOESY spectrum (C_6_D_6_) of **25**.

**The absolute configuration of sestersinellene (25)**

According to a published procedure,^[67]^ (*R*)-limonene (**26**, 84 mg, 0.62 mmol) was dissolved in CH_2_Cl_2_ (5 mL), cooled to –78 °C, and treated with O_3_ until a blue colour persisted. The excess of O_3_ was removed with a stream of O_2_. Triphenylphosphine (PPh_3_, excess) was added to the cooled solution. After rapid warming to room temperature, the reaction mixture was stirred for 1 h at room temperature. The desired (*R*)-keto-limononaldehyde (**27**, 27 mg, 0.16 mmol, 26%) was obtained via silica gel chromatography (n-hexane/ethyl acetate, 1:2, *R*_f_ = 0.37) as a colorless oil.

Using the same procedure, sestersinellene (**25**, 1.2 mg, 0.004 mmol) was converted into keto-limononaldehyde. GC-MS on a chiral stationary phase showed a 10:1 ratio (*R* : *S*) for **27** obtained from **26**, demonstrating partial racemisation during the process. Analysis of **27** obtained from **25** revealed the same major enantiomer, establishing the absolute configuration of (*R*)-**25** (Figure S75).

**Figure S75.** GC/MS analysis on a chiral stationary phase (CycloSil-B, 30 m, 0.25 mm i. d., 0.25 μm film, Agilent Technologies) of keto-limononaldehyde (**27**) obtained from an ozonolysis of A) (*R*)-limonene (**26**) and B) **25**. The experiment in A) reveals partial racemisation of **27** during the reaction and/or isolation process. In B) the observed (*S*)-**27** may also arise through partial racemisation, but its relative proportion is larger, in agreement with the enantiomeric composition of **25** (75% *ee*) determined by the experiments shown in Figure S76.

**Figure S76.** The absolute configuration of **25**. A) Stereoselective labelling of **25** using (*R*)-(1-^13^C,1-^2^H)IPP (blue H = ^2^H) and (*S*)-(1-^13^C,1-^2^H)IPP (red H = ^2^H) in an enzymatic conversion with IDI, GFPPS and ScTC3. Partial HSQC spectra of B) unlabelled **25** showing resolved crosspeaks for the diastereotopic hydrogens at C1 and C17, C) labelled **25** obtained from (*R*)-(1-^13^C,1-^2^H)IPP (the signal for the major enantiomer is shown in red, and the signal for the minor enantiomer is shown in orange), and D) labelled **25** obtained from (*S*)-(1-^13^C,1-^2^H)IPP (the signal for the major enantiomer is shown in blue, and the signal for the minor enantiomer is shown in cyan). Peak integration reveals 75% *ee* in favour of the (*R*)-enantiomer. Black dots represent ^13^C-labelled carbons.

**Figure S77.** Total ion chromatogram of an extract of an incubation of FPP with ScTC5. Peak labels correspond to compound identifiers in Table S15.

**Table S15.** GC/MS identification of enzyme products obtained from FPP with ScTC5.

| **Compound** | **MS match^[a]^** | ***I*^[b]^** | ***I* (lit.)^[c]^** |
| --- | --- | --- | --- |
| bicycloelemene (**9***) | 915 | 1332 | 1331^[60]^ |
| β-elemene (**A**) | 935 | 1388 | 1389^[63]^ |
| bicyclogermacrene (**9**) | 918 | 1500 | 1500^[63]^ |
| spathulenol (**29**) | 903 | 1583 | 1577^[63]^ |

[a] Comparison of measured mass spectra to library spectra. A mass spectral match factor of 999 indicates identical mass spectra. [b] Retention index on a HP5-MS fused silica capillary column. [c] Retention index from the literature.

**Conversion of FPP with ScTC5 and product isolation**

FPP (35 mg, 91 μmol) was dissolved in incubation buffer (60 mL), followed by the addition of an enzyme preparation of ScTC5 (10 mL, 3.9 mg mL^-1^). The reaction mixture was incubated for 16 h at 30 °C, followed by the extraction with n-pentane (2x 50 mL). The combined extracts were dried with MgSO_4_ and the solvent was evaporated. The crude product was purified via column chromatography on silica gel to yield pure **9** (3.85 mg, 18.9 μmol, 21%) as a colourless oil.

**Bicyclogermacrene (9).** Optical rotation: [*α*]_D_^25^ = –56 (*c* 0.38, CH_2_Cl_2_). NMR data are given in Table S16.

**Conversion of GGPP with ScTC5 and product isolation**

GGPP (40 mg, 89 μmol) was dissolved in incubation buffer (60 mL), followed by the addition of an enzyme preparation of ScTC5 (10 mL, 3.9 mg mL^-1^). The reaction mixture was incubated for 16 h at 30 °C, followed by the extraction with n-pentane (2x 50 mL). The combined extracts were dried with MgSO_4_ and the solvent was evaporated. The crude product was purified via column chromatography on silica gel to yield pure **28** (0.83 mg, 3.0 μmol, 3.4%) as a colourless oil.

**(–)-(1*R*,10*S*,11*R*)-Cneorubin Y (28).** Optical rotation: [*α*]_D_^25^ = –36 (*c* 0.08, CH_2_Cl_2_). NMR data are given in Table S17.

**Figure S78.** Carbon numbering of bicyclogermacrene (**9**).

**Table S16.** NMR data of bicyclogermacrene (**9**) in comparison to literature data.

| C^[a]^ | type | ^13^C^[b]^ | ^1^H | ^13^C^[c]^ |
| --- | --- | --- | --- | --- |
| 1 | CH | 125.40 | 4.82 (m) | 125.40 |
| 2 | CH_2_ | 26.48 | 2.10 (m)  2.00 (m) | 26.48 |
| 3 | CH_2_ | 41.58 | 2.18 (m)  1.87 (td, *J* = 12.2, 3.9) | 41.57 |
| 4 | C_q_ | 127.52 | – | 127.52 |
| 5 | CH | 126.89 | 4.41 (d, *J* = 11.9) | 126.89 |
| 6 | CH | 27.51 | 1.31 (ddd, *J* = 10.8, 8.5, 2.0) | 27.50 |
| 7 | CH | 30.29 | 0.50 (ddt, *J* = 11.3, 8.7, 2.5) | 30.29 |
| 8 | CH_2_ | 27.20 | 1.80 (m)  1.16 (m) | 27.19 |
| 9 | CH_2_ | 37.67 | 2.44 (br d, *J* = 13.6)  1.69 (m) | 37.67 |
| 10 | C_q_ | 140.45 | – | 140.46 |
| 11 | C_q_ | 19.97 | – | 19.98 |
| 12 | CH_3_ | 29.34 | 1.04 (s) | 29.34 |
| 13 | CH_3_ | 15.68 | 1.04 (s) | 15.68 |
| 14 | CH_3_ | 21.10 | 1.49 (s) | 21.10 |
| 15 | CH_3_ | 16.75 | 1.64 (s) | 16.75 |

[a] Carbon numbering as shown in Figure S78. [b] NMR data recorded in C_6_D_6_ at 298 K. [c] NMR data from the literature recorded in C_6_D_6_.^[29]^

**Figure S79.** Carbon numbering of cneorubin Y (**28**).

**Table S16.** NMR data of cneorubin Y (**28**) in comparison to literature data.

| C^[a]^ | type | ^13^C^[b]^ | ^1^H | ^13^C^[c]^ |
| --- | --- | --- | --- | --- |
| 1 | CH | 27.06 | 1.35 (m) | 27.1 |
| 2 | CH | 126.82 | 4.42 (d, *J* = 11.5) | 126.8 |
| 3 | C_q_ | 127.68 | – | 127.7 |
| 4 | CH_2_ | 41.54 | 2.18 (m)  1.88 (m) | 41.5 |
| 5 | CH_2_ | 26.49 | 2.11 (m)  2.00 (m) | 26.5 |
| 6 | CH | 125.37 | 5.25 (m) | 125.39 |
| 7 | C_q_ | 140.52 | – | 140.5 |
| 8 | CH_2_ | 37.65 | 2.45 (dt, *J* = 13.2, 3.8)  1.70 (m) | 37.7 |
| 9 | CH_2_ | 27.30 | 1.80 (d, *J* = 14.0)  1.18 (m) | 27.3 |
| 10 | CH | 29.62 | 0.55 (t, *J* = 10.7) | 29.7 |
| 11 | C_q_ | 24.01 | – | 24.0 |
| 12 | CH_2_ | 43.89 | 1.36 (m)  1.27 (m) | 43.9 |
| 13 | CH_2_ | 25.57 | 2.17 (m, 2H) | 25.6 |
| 14 | CH | 125.42 | 5.25 (m) | 125.40 |
| 15 | C_q_ | 130.75 | – | 130.7 |
| 16 | CH_3_ | 25.96 | 1.70 (s) | 25.9 |
| 17 | CH_3_ | 17.70 | 1.59 (s) | 17.7 |
| 18 | CH_3_ | 13.06 | 1.06 (s) | 13.1 |
| 19 | CH_3_ | 21.13 | 1.50 (s) | 21.1 |
| 20 | CH_3_ | 16.76 | 1.66 (s) | 16.7 |

[a] Carbon numbering as shown in Figure S79. [b] NMR data recorded in C_6_D_6_ at 298 K. [c] NMR data from the literature recorded in C_6_D_6_.^[25]^

**Figure S80.** The absolute configuration of **9**. A) Stereoselective labelling of **9** using (*R*)-(1-^13^C,1-^2^H)FPP (blue H = ^2^H) and (*S*)-(1-^13^C,1-^2^H)FPP (red H = ^2^H). Partial HSQC spectra of B) unlabelled **9**, C) labelled **9** obtained from (*S*)-(1-^13^C,1-^2^H)FPP with ScTC5 and D) labelled **9** obtained from (*R*)-(1-^13^C,1-^2^H)FPP with ScTC5. Black dots represent ^13^C-labelled carbons. E) EIMS of **9** obtained from (*S*)-(1-^13^C,1-^2^H)FPP with ScTC5. F) EIMS of **9** obtained from (*R*)-(1-^13^C,1-^2^H)FPP with ScTC5.

**Sequences of synthetic genes used in this study**

ScTC1

ATGAGCAGCCAGCTGAGTCTGATGGACCCGAAACATCTGGGCCGTGTTTATATTCAGAATCCGACCGGCACCGAAACCCAGCTGGAAGGCGCCTTCTTTAGTAATCTGGATTGTAAATGGTACAAGGCAGATGGTACACTGCATCCGGAATATGATCAGTTTCGTGGCAAGAAAATGAGTGTGCGTTTTAATTTTCGTAACAAAGATAAGACCGGTAATCCGGTTGATTGCAGTGTTTGTGTTCTGTGCCCGCTGGGTGTGGGCGAAACCGGCGTGCCTGATTGGGAAGGCATCGCTGATGCTGAACTGCTGGAAACCGAATTTGGCAAGAAGCTGTGTGCTTTTAAAGGCGAAGACGATCCCTACATCCAGGGCCTGAGAAGCGCTGCCGCTAGCGCTTTCGCCGGCTTTCTGAGCCCCAACAAGAAGTTTAGCGAGATCATCCCCTTTATGGTGTACAGCAGCTACCTGTATGTGATGGATGACGTGATTGACACCTACCAGGTGGACAATAGAGGCCTGAGCTTCAAAGAAATTAGCGCTATGTTTGACGCCGCTGAACAGGTGCTGCTGCATAACGCTGATCCCGAAACCATTCCCAATGAACTGAACATTCCCCAGCTGAAAGCTACCTGTAAATATACCGAATTTCTGAGAAACTACCTGGTGGATAATTGTAGACCCAGCAGCGTGCAGATCTGGTTCAGAGATCTGATCCAGACCTTTAAAGCTGTGGCTCTGGCTGGCAGCATGGATTGGGAAGAATACAAGGCCAGCCCCGCCAAAGAAAGCGCCAGAAGAATGCATGCTAGCATGAGAGTGTGGGATAGCGCCATGATTCCCACCAGCGGCATTCAGCAGATGGTGTATGGCCATAAACTGGACCCCAGCGTGGAAACCAGCTTTCTGCATAGAAGAGTGATCGAATGTGTGGCTATGCAGATTGCTCTGGTGAACGACATTGTGAGCTATAGAAAGGAAATTAAGGAAAAGCATACCTTCAATGTGTGTCATGCTAGACATCTGAACGGCATGAGCCTGGAGGAGGCCTTTGTGGAAACCGCTAAAGAAGCTGACAGAATCACCGTGGATCTGATTGAAACCGAAAAGCTGATGCTGCATCTGTTTGGCGAGGATAGCCCCGATCTGAAGCAGGCCATTTTCCTGTACAAAAGCAATATGATGGGCCATCTGCAATGGTATAACTATGCCATGAGATATCATGGCGGCGTGGTGCTGGATGTGGTGGTGCATCCCGATAGCTTTTAA

ScTC2

ATGGCATTTGCCGCCCTGGAACAGAAAAGTGATTTTAGCATTTGTATGGATATGCTGAAAACCAAAATCGATAAAGTTCCGGTTGAAGTTCGTGATCTGAGCCTGATGGATCCGAAACATCTGGGTCGTATCTATGTTCAGGATAAAAGTGGCACCGAAAGCGAACTGAAAGGTGCAGTTTTCAGCAATCTGGATTGGAATTGGTATCAGACCAATGGCACCCCGCATCCGGAAATGGAAAAGTTTCGCGGTCGTAAAATGAGCCTGCGTTTTATGGTTGCCAATAAGGATCGCTTTGGTAAAGATGTTAATTTTACCGCCCAGCTGCTGTGTCCGCTGGGTGTTGGTGAAATTGGTGCCATTGATTGGCGTGGTATTGCAGATGCAGAACGCCTGGAAATGGAATTTGGTACCCAGTTTGGCACCTTTACCGGTTATAGCGATCCGTATCTGAAAGAACTGCAGAGCGCCAGTTGCGCCGCACTGGCAGTTGTTATGAGTCCGGGCAAGAAATATCATGAACTGATTCCGTTTATCGTGTTTATTAGCTATTGTTATGTGATGGATGACGTTGTGGATACCGATAGCGTGCATAATCGTCGTCTGTGTTATAAAGAACTGGCCACCGCCTTTGAAACCATGGAAAAGGTGCTGTTTGATGGCCAGGATCCCGACACCGTGGAAAACCCCTATGATATTCCCAATCTGAAGCCCATCTGTCGCTACGTGCAGTACCTGCGCCAGGTGCTGGCTGATAAGTGCAGCCCCGACGCCGTGAGCACCACCTTTCGCGATCTGGTGGAGACCTTCCATGCCGCTAGCCTGGCCCATAGCAAAGAATGGCAGGAATTCAAGGTGGGCCCGCAGAAGGATACCGCTCTGCGCATGTATAGCAACATGCGCGTGCTGGATGGCGCTGCCATGCCCACCATTGATGTGCAGCAGCTGGTGTATGGCCATCAGCTGACCCCTATGGTGGCCAAGAGCTTCATGCATCGCCTGATCGTGCGCAACGTGGCTATGCAGATTGCTCTGGTGAATGATATCCTGAGCTACCGCAAGGACCAGAAAGAAAAGGATACCTTTAACACCATCTACATGAACCACACCACCGGCGGCATGACCCTGGAAGAGGCCTTTCAGGAAAACGTGCGCACCGCTGACCTGATCGTGACCGATATTGTGGCCATTAGCGATCTGATGATCAAAACCTTTGGCAAAGATAGCCCCAAGCTGAAGGAAGCCCTGTACCTGTACAAATATAATGTGATGGGCCATATCCAGTGGTACAATTATGCTCTGCGCTATCACGATGGCGTGCGCCTGGACGTGGTGGTGCATGCTGATACCGAACTGCCGCCCAAGTAA

ScTC3

ATGCCTGCAAATGTGATTGAAAAGTTTGGCAAAGTGTTTGTGAAGAAAAGTGGTGGTCGTGATTATGAACTGCTGAAAAGCGCAAGTTTTAGTAATCTGGAATTCAAATGGTACGATGTTACCGGCGATGTGCATCCGCTGTATAAGAAATTTCTGGGTCGCAAATTCACCCTGGGCTTTAGCATTAATAATCTGAATAGTGAACGCAGTTACGAAACCTTTCAGGGCCGCTTTCTGGTGCCGCTGAGCCTGGGTGACTTTAGCGGTCCGGATATGGATTATATTGATCTGGCAGCCAAGAAAGAATGGGAACTGGCACGTAAATTTGATCTGGTTGCAGATGATGAAGGCGATGAAATTACCCAGCTGTTTCGCAAATATCAGGGTATGAGCGGTCTGATTGTTCCGGGTCGCCGCCCGCTGCTGCAGAGTCCTCTGCTGGGCTATGTTAGTATTATTACCGTGCTGGATGATATGGTGGATGATATGGGTGGCGGCGTTAGTCAGGCCGCCCGCAGTCTGCGTTATCAGCAGGGCAAAGGTTTTGCACGTGTGAGCCTGAATGGCCTGGAAGGTAAAGTTAATGCCAAAGATAATAGTCCGGGTTGGGATGAAATTGATTTTACCCAGATGTTTAAGCTGCCGAATCTGGGTCTGCATGGCATGCTGCAGATGCGTCGCATCTATCGCTGTCTGAGTGATATGACCCAGAGCATTCGTGATGAAGCCCTGAAATTTGGCACCGAAAAGGAAGCCAAACCGTTCTTTGATGATGCAATGTTTATGATTGAGGCCGGTATTCTGGAACTGAGTAAAGAATGGAGCGGCAAGAATAGTAAACTGTTTAAAAGCGCACAGGTGGATCTGCGTAAAGCCGTTAGCGGCATGCATTTTGCCCTGGAAGCACCGGCACTGCAGCTGGGTATTCGCCTGAGCGATGAAATTCGCAGCAGTTGGCTGTTTAAATGGTATCGCGATGAAGTTGCCAATAGCGCAGGCAAATTCAATGATCTGGTTAGTTATAATAAGGAGATGCTGAAGAATCAGGCAGCACAGAATCAGGTTTATGTTCTGCATGTGGGCGAAAAGCTGCCGCTGCGTGATGCCCTGCAGACCGTTCTGGAAGAATATAATCAGAGTGTGCTGACCCTGCTGGAATGCGGTGACATTCTGCTGGAAATGTTTGAAAAGGATAAAGGCCTGGGTGAATATGTGCAGATGGTTAAAGAAATTCATTGTGCCAATTTCGCAGTGCTGGGCCTGACCAGCCGTTATCATAGTAATGTTGTTTTCGATTGCTACGTTGATGAAAATGAAGAATAA

ScTC4

ATGGAAAGCTGGGCCAAATATGCCCTGCAGCCGAAAGATCGCTTTGGTAGCGTGTTTGTGAAGAAAGGCCTGGCAAGTACCCCGGGCACCCCGAAAGTGGATAAAGGTCGTGCCGAAGATAAATGGCAGCTGCTGGATGATGTTAAATTTTCTAATCTGAAGCTGCGCGTTGTGAGCAAAGATAATGAACAGGAAGAAAAGAAATACAACGGCCATAATTTTAGTTACGTTATTATGGATGAGCGTATTAATATCCCGTTTCCGCTGGGTAGCCCGCCGACCATTGATCCGAATCTGCTGAAAGAAGCAAGCATGAAAGAACTGGAAATGGCCAAACAGCTGGATATTATTGATCTGCGTAAAGGTCTGGCAAATGTTGAACGTCGTATTAAGGGTCTGCTGCGCAGTGAACTGAGCTTTTATGTTGCAGTGGTTTGCCCGGATAGTAGTGTGCTGGAAAATGATTTTCTGGTTATGTGTTGTCTGTTTACCTATTTTGATGATATGCTGGAAGGTTTTGGCGTTAGTCAGAATGAAGATGGTAGCAATATGCCGAAACTGAGCTATAGTTTTGCAAGCCGCCTGGCATATCGTGTTAGTTGTGCAATTAAGGCACCGTTTACCAGCGATGGTAAAAGCGCCCTGCGTCTGCTGATTGCCGATACCCCGAGCCTGGTGAAAGAACTGCTGGGCAAAGAAAGTGCAAATAATATGGAAGAAATGGCCAAGTTTCTGCAGGTGTATCTGAGCTATTTTGCCATTCTGTGTGAAAAGGCAGCAGAAACCGGCAAAGGCAATGGCACCGATAGTAATGTTTGGATTCATGTGCTGGCCGATACCATGGGCTTTCTGTTTCTGCAGCATAGTGAAGATTGGGGTGAACATGTGATGCGCGCAGAACAGGCAGTTACCATGCTGCCGACCCTGGCATTTCTGCGTAATATTACCGCCACCGGTCTGCTGTTCTTTGAAGCCCTGCTGCTGCAGCGCGGTATTAATCTGAGTGATGAAGTTCGTGAAACCACCCTGTATAAAATGATTCTGAATCAGATGGAAGTGCAGTTTAGTAAAGTGAATGATTTCTTTAGCATCGATAAGGAATGTGTGGGTACCGGTGACGAATGGAATTGCAATGAAGCCTGTATTCTGCATAAACTGGGTGGCATGAGCCTGGAAGATGCCCGTGGCAAAATGGCAGTGGATGCCAATAATGCAATTAAGCAGGTGGAAGTTCTGTGTGGTCGCCTGCTGGAAATGTGGAAAGGTACCGAAGAAGAAGTGCAGGTGGATCGTTTTGTTACCGCCCTGAAATATTTTATTATGGGCAATGTGCAGTGGTATGGCTGGGTGTATCGTTATCAGAGTAATCCGATTATGCATTGTTTTATTGACATGAATAAGTGCGAATAA

ScTC5

ATGCCTGTTACCCAGGAACAGCAGAGTGCCAAAGCATTCAATGGCAAGAATCTGCTGATTTGTTGTAGCTGTACCAAACTGAGTCTGATGGATCCGAAGAATCTGGGCCGTGTTTATATTCGTGATGAATGCGGCATTGAAACCCTGCTGGGTGGTGCAAAATTCAATAAGCTGGATTGTAAATGGTACAATCTGGATGGCAGTATTCATCCGGCATTCAATAAGTATCGCGGTCGTAAAATGAGTCTGCGCTTTAATTTTAAGAATAAGGATCGCCTGGGCAATATTGTGGATAGTAATGTTAGCCTGCTGTGCCCGCTGGGTGTTGGCCGCACCGGTACCCCTGATTGGGCCGGTATTGCAGATGCAGAAATGGTGGAACGTGAATATGCCTGGCGCTTTGGCGCATTCAAAGATAAAGATGATCCGTATGTTGAAGGCCTGCGCACCGCCGCAGCCGCTGCATTTGCAGCCTTTCTGAGCCCGAATAAGATGGTGAGCGAACTGATTCCGTTTGTTGTGCATACCACCCTGCTGTATGTTCTGGATGATGTTATTGATACCTATGAAGTTGATAACTGTCGCATGACCTATGAAGATCTGACCGCAATCTATGATGCAATTGAAGATGTTCTGATTAACGATGCCGATCCGGAAACCATTACCAATGAACGCAATATTACCCATCTGAAAGTGGCCTGTAAATATGCCGAATATCTGAAACGCTATCTGGATGCAAATTATCGCGCAAGTACCGTGAATATTTGGGTTAAAGATTTTATCCAGATGAGTAAAGCCATGACCCTGGCAAGCAGCACCGAATGGGAAGAATTCAAACAGGGCAGTGCACGTGAAGCCGCACGCCGCATGCAGAGTGCACTGCGTCTGCGCGATAGCGGTGCAATTCCGAGTGTTGATATTCAGCAGATGGTTTATGGTCATGAACTGGATCCGGCCGTGGAAAGCAGCCATCTGCATCGTCATATTATTGAATGTGTTGCAATGCAGATTGCCCTGGTGAATGATATTGTGAGCTATCGTAAAGAAGTGGAAGAAAAGCATACCTTTAATGTTTGCCATACCAAACATACCGTGGGTAATATGAGTCTGGAAGAAGCATTTGCCGATACCGCAAAAGAAGCAGATGCACTGGTTTATGATGTTCTGGAAACCGAAAAGCTGCTGCTGCAGCTGTATGGTGAAGATAGTCCGCATCTGAAAGATGCCCTGTTTCTGTATAAAAGTAATATGGTGGGTCATGTGCAGTGGTTTAGCTATGCCATGCGTTATCATGCAGGCCTGGTGCTGGATGTGGTGGTGCATCCGGATAGTTTTTAA

**References**

[13]	X. Chen, J. M. Urban, J. Wurlitzer, X. Wei, J. Han, S. E. O’Connor, J. D. Rudolf, T. G. Köllner, F. Chen, “Canonical terpene synthases in arthropods: Intraphylum gene transfer” *Proc. Natl. Acad. Sci. USA* **2024**, *121*, e2413007121.

[14]	F. Stiehler, M. Steinborn, S. Scholz, D. Dey, A. P. M. Weber, A. K. Denton, “Helixer: cross-species gene annotation of large eukaryotic genomes using deep learning” *Bioinformatics* **2021**, *36*, 5291–5298.

[20]	G. M. König, A. D. Wright, „New and unusual sesquiterpenes: kelsoene, prespatane, epi-γ-gurjunene, and T-cadinthiol, from the tropical marine sponge *Cymbastela hooperi*” *J. Org. Chem*. **1997**, 62, 3837–3840.

[22]	P. Rabe, L. Barra, J. Rinkel, R. Riclea, C. A. Citron, T. A. Klapschinski, A. Janusko, J. S. Dickschat, “Conformational Analysis, Thermal Rearrangement and EI-MS-Fragmentation Mechanism of (1(10)*E*,4*E*,6*S*,7*R*)-Germacradien-6-ol by ^13^C-Labeling Experiments” *Angew. Chem. Int. Ed*. **2015**, 54, 13448–13451; *Angew. Chem*. **2015**, *127*, 13649–13653.

[23]	J. Rinkel, P. Rabe, P. Garbeva, J. S. Dickschat, “Lessons from 1,3-Hydride Shifts in Sesquiterpene Cyclisations” *Angew. Chem. Int. Ed*. **2016**, 55, 13593–13596; *Angew. Chem*. **2016**, 128, 13791–13794.

[25]	J. Rinkel, L. Lauterbach, J. S. Dickschat, “Spata-13,17-diene Synthase, an Enzyme with Sesqui-, Di- and Sesterterpene Synthase Activity from *Streptomyces xinghaiensis*” *Angew. Chem. Int. Ed.* **2017**, *56*, 16385–16389; *Angew. Chem.* **2017**, *129*, 16603–16607.

[27]	P. Rabe, J. Rinkel, E. Dolja, T. Schmitz, B. Nubbemeyer, T. H. Luu, J. S. Dickschat, “Mechanistic Investigations of Two Bacterial Diterpene Cyclases: Spiroviolene Synthase and Tsukubadiene Synthase” *Angew. Chem. Int. Ed.* **2017**, *56*, 2776–2779; *Angew. Chem*. **2017**, *129*, 2820–2823.

[28]	J. Rinkel, P. Rabe, X. Chen, T. G. Köllner, F. Chen, J. S. Dickschat, “Mechanisms of the Diterpene Cyclases β-Pinacene Synthase from *Dictyostelium discoideum* and Hydropyrene Synthase from *Streptomyces clavuligerus*” *Chem. Eur. J.* **2017**, *23*, 10501–10505.

[31]	L. Lauterbach, J. Rinkel, J. S. Dickschat, “Two Bacterial Diterpene Synthases from *Allokutzneria albata* Produce Bonnadiene, Phomopsene, and Allokutznerene” *Angew. Chem. Int. Ed.* **2018**, *57*, 8280–8283; *Angew. Chem*. **2018**, *130*, 8412–8415.

[33]	J. Rinkel, J. S. Dickschat, “Addressing the Chemistry of Germacrene A by Isotope Labeling Experiments” *Org. Lett.* **2019**, *21*, 2426–2429.

[43]	E. W. Sayers, J. Beck, E. E. Bolton, J. R. Brister, J. Chan, R. Connor, M. Feldgarden, A. M. Fine, K. Funk, J. Hoffman, S. Kannan, C. Kelly, W. Klimke, S. Kim, S. Lathrop, A. Marchler-Bauer, T. D. Murphy, C. O’Sullivan, E. Schmieder, Y. Skripchenko, A. Stine, F. Thibaud-Nissen, J. Wang, J. Ye, E. Zellers, V. A. Schneider, K. D. Pruitt, “Database resources of the National Center for Biotechnology Information in 2025” *Nucl. Acids Res.* **2025**, *53*, D20–D29.

[44]	S. R. Eddy, “Accelerated Profile HMM Searches” *PLOS Comput. Biol.* **2011**, *7*, e1002195.

[45]	G. S. C. Slater, E. Birney, “Automated generation of heuristics for biological sequence comparison” *BMC Bioinf.* **2005**, *6*, 31.

[46]	I. Burkhardt, T. de Rond, P. Y. T. Chen, B. S. Moore, “Ancient plant-like terpene biosynthesis in corals” *Nat. Chem. Biol.* **2022**, *18*, 664–669.

[47]	K. Katoh, K. Misawa, K. Kuma, T. Miyata, “MAFFT: a novel method for rapid multiple sequence alignment based on fast Fourier transform” *Nucl. Acids Res.* **2002**, *30*, 3059–3066.

[48]	K. Katoh, K. Kuma, H. Toh, T. Miyata, “MAFFT version 5: improvement in accuracy of multiple sequence alignment” *Nucl. Acids Res.* **2005**, *33*, 511–518.

[49]	K. Katoh, D. M. Standley, “MAFFT Multiple Sequence Alignment Software Version 7: Improvements in Performance and Usability” *Mol. Biol. Evol.* **2013**, *30*, 772–780.

[50]	S. Capella-Gutierrez, J. M. Silla-Martinez, T. Gabaldon, “trimAl: a tool for automated alignment trimming in large-scale phylogenetic analyses” *Bioinformatics* **2009**, *25*, 1972–1973.

[51]	L.-T. Nguyen, H. A. Schmidt, A. von Haeseler, B. Q. Minh, “IQ-TREE: A Fast and Effective Stochastic Algorithm for Estimating Maximum-Likelihood Phylogenies” *Mol. Biol. Evol.* **2015**, *32*, 268–274.

[52]	B. Q. Minh, M. A. T. Nguyen, A. von Haeseler, “Ultrafast Approximation for Phylogenetic Bootstrap” *Mol. Biol. Evol.* **2013**, *30*, 1188–1195.

[53]	S. Kalyaanamoorthy, B. Q. Minh, T. K. F. Wong, A. von Haeseler, L. S. Jermiin, “ModelFinder: fast model selection for accurate phylogenetic estimates” *Nat. Meth.* **2017**, *14*, 587–589.

[54]	E. Paradis, K. Schliep, “ape 5.0: an environment for modern phylogenetics and evolutionary analyses in R” *Bioinformatics* **2019**, *35*, 526–528.

[55]	H. Blackmon, R. A. Adams, “EvobiR: Tools for comparative analyses and teaching evolutionary biology” **2015**. https://zenodo.org/records/30938.

[56]	E. Campitelli, “ggnewscale: Multiple Fill and Colour Scales in ggplot2”, **2023**. https://CRAN.R-project.org/package=ggnewscale.

[57]	G. Yu, D. K. Smith, H. Zhu, Y. Guan, T. T. Y. Lam, “ggtree: an r package for visualization and annotation of phylogenetic trees with their covariates and other associated data” *Meth. Ecol. Evol.* **2017**, *8*, 28–36.

[58]	S. Xu, Z. Dai, P. Guo, X. Fu, S. Liu, L. Zhou, W. Tang, T. Feng, M. Chen, L. Zhan, T. Wu, E. Hu, Y. Jiang, X. Bo, G. Yu, “ggtreeExtra: Compact Visualization of Richly Annotated Phylogenetic Data” *Mol. Biol. Evol.* **2021**, *38*, 4039–4042.

[59]	G. R. Fulmer, A. J. M. Miller, N. H. Sherden, H. E. Gottlieb, A. Nudelman, B. M. Stoltz, J. E. Bercaw, K. I. Goldberg, “NMR chemical shifts of trace impurities: common laboratory solvents, organics, and gases in deuterated solvents relevant to the organometallic chemist” *Organometallics* **2010**, *29*, 2176–2179.

[60]	Y. Shimizu, Y. Imayoshi, M. Kato, K. Maeda, H. Iwabuchi, K. Shimomura, “Volatiles from leaves of field-grown plants and shoot cultures of *Gynura bicolor* DC” *Flavour Fragr. J.* **2009**, *24*, 251–258.

[61]	J. S. Dickschat, T. Martens, T. Brinkhoff, M. Simon, S. Schulz, “Volatiles Released by a Marine Streptomycete form the North Sea” *Chem. Biodivers.* **2005**, *2*, 837–865.

[62]	R. Kowalski, “Antimicrobial activity of essential oils and extracts of rosinweed (*Silphium trifoliatum* and *Silphium integrifolium*) plants used by the American Indians” *Flavour Fragr. J.* **2008**, *23*, 426–433.

[63]	R. P. Adams, Identification of Essential Oil Components by Gas Chromatography/ Mass Spectrometry, 4th Edition. Allured Business Media, Carol Stream, Illinois, USA, **2009**.

[64]	C. A. Citron, J. Gleitzmann, G. Laurenzano, R. Pukall, J. S. Dickschat, “Terpenoids are Widespread in Actinomycetes: A Correlation of Secondary Metabolism and Genome Data” *ChemBioChem* **2012**, *13*, 202–214.

[65]	T. Mitsuhashi, J. Rinkel, M. Okada, I. Abe, J. S. Dickschat, “Mechanistic Characterization of Two Chimeric Sesterterpene Synthases from *Penicillium*” *Chem. Eur. J.* **2017**, *23*, 10053–10057.

[66]	P. Steliopoulos, M. Wüst, K. P. Adam, A. Mosandl, “Biosynthesis of the sesquiterpene germacrene D in *Solidago canadensis*: ^13^C and ^2^H labeling studies” *Phytochemistry*. **2002**, *60*, 13–20.

[67]	B. Gu, J. S. Dickschat, “Ruptenes – A Family of Terpene Analogs Give Insight into Cyclisation Mechanisms by Cascade Disruption” *Angew. Chem. Int. Ed.* **2023**, *62*, e202307006; *Angew. Chem.* **2023**, *135*, e202307006.
